# Polyethylene Microplastics Inhibit Peanut Nodulation via Metabolic and Transcriptional Pathways

**DOI:** 10.3390/plants15060915

**Published:** 2026-03-16

**Authors:** Yue Wu, Zhengfeng Wu, Yongmei Zheng, Jishun Yang, Jiancheng Zhang, Hongfeng Wang, Tianyi Yu, Juxiang Wu, Shangxia Li

**Affiliations:** Shandong Peanut Research Institute, Qingdao 266100, China; ymzhengrice@163.com (Y.Z.); jsyang94@126.com (J.Y.); jianch-zh@hotmail.com (J.Z.); wanghf@sdu.edu.cn (H.W.); tianyi_1984@126.com (T.Y.); wujuxiang2026@163.com (J.W.); lishangxia@hotmail.com (S.L.)

**Keywords:** microplastic pollution, legume–rhizobia symbiosis, impaired nodulation ability, soil contamination, peanut transcriptome

## Abstract

Polyethylene (PE) microplastics (MPs) from residual mulch films are prevalent in peanut-cultivated soils, yet their specific effects on peanut nodulation remain unclear. This study investigated the impacts of PE-MPs at concentrations of 0.2%, 0.6%, and 1.0% on peanut nodulation. Results indicated that PE-MPs significantly reduced peanut nodule number. Transcriptome analysis revealed that all three concentrations of PE-MPs down-regulated nodulation-related flavonoids, promoted lignin deposition in cell walls, disrupted antioxidant system, and enhanced the accumulation of antimicrobial substances, collectively impairing peanut nodulation efficiency. These findings indicate that PE-MPs substantially compromise the symbiosis between peanut and rhizobia, and provide insights into their interference with plant–beneficial microbe interactions in contaminated soils.

## 1. Introduction

Microplastics (MPs) are pervasive contaminants in terrestrial ecosystems [1]. Soils, particularly agricultural systems, have been identified as the primary reservoirs of MPs due to cumulative inputs from anthropogenic activities [2]. Researchers have found that MPs enter farmland through various agricultural activities, such as the application of plastic mulches, the usage of sewage sludge or wastewater, etc. [3,4]. Over time, the accumulated MPs in the soil pose a threat to the sustainability of an agroecosystem, adversely affecting plant growth and development, microbial community structure and function, and even human health [5].

Polyethylene MPs (PE-MPs) have become the predominant contaminants in agricultural soils, primarily originating from the degradation of widely used PE mulch films [6]. Current studies have demonstrated that PE-MPs disrupt diverse physiological processes in crops, such as maize (*Zea mays*), cucumber (*Cucumis sativus*), and lentil (*Lens culinaris*), including growth regulation, photosynthetic efficiency, metabolic pathways, and defense mechanisms [7,8,9]. Further evidence indicates that PE-MPs alter the expression of genes involved in phenylpropanoid biosynthesis and sugar transport pathways in maize, ultimately leading to growth inhibition [10]. Additionally, PE-MPs also affect the structure and function of crop rhizosphere microbial communities. For instance, PE-MPs have been shown to increase total microbial abundance and enrich PE-degrading microbial community, while reducing populations of nitrogen (N)-cycling bacteria in crop rhizosphere soil [11,12]. Despite the effects of PE-MPs on individual plant physiology and rhizosphere soil microbiology already documented, critical knowledge regarding the impact of PE-MPs on plant–microbe symbiotic interaction remains unknown. This oversight is particularly significant given the fundamental role of plant–microbe interactions in maintaining agricultural ecosystem function and crop productivity.

Peanut (*Arachis hypogaea* L.) is an important leguminous crop with substantial economic value, widely cultivated in tropical and subtropical regions. Throughout their growth cycle, peanuts form nodules by symbiosis with rhizobia, thereby obtaining the nitrogen they require through the process of biological nitrogen fixation [13]. This natural process provides a valuable mechanism for reducing agricultural reliance on synthetic nitrogen fertilizers [9]. At present, due to the widespread use of PE mulch films in peanut cultivation, plants face serious threats from PE-MPs. Previous research has documented the effects of PE-MPs on peanut growth, nodulation, rhizosphere microbiome composition, and N assimilation [3]. However, the mechanism by which PE-MPs influence peanut nodulation is not yet understood, and this critical knowledge gap limits a comprehensive understanding of the ecological risks of MP pollution in agricultural systems. Therefore, this study used transcriptomic analysis to elucidate the mechanisms through which different concentrations of PE-MPs affect peanut–rhizobia symbiotic nodulation. We hypothesized that PE-MPs inhibit peanut nodulation, likely by altering metabolic pathways and defense responses associated with the nodulation process. This study is the first to systematically reveal the molecular mechanisms by which PE-MPs interfere with legume–rhizobia symbiosis at the transcriptional level.

## 2. Results

### 2.1. Effects of PE-MPs on Peanut Nodulation

**Figure 1:**
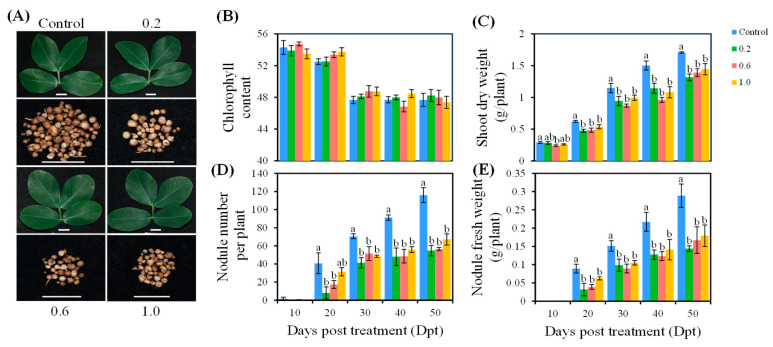
Effects of PE-MP concentration on peanut plants over time. (**A**) Leaves and nodules at 40 days post-treatment (dpt). (**B**–**E**) Dynamics of chlorophyll content (**B**), shoot dry weight (**C**), nodule number per plant (**D**), and nodule fresh weight (**E**) measured at 10, 20, 30, 40, and 50 dpt. Control: no PE-MP addition; 0.2: 0.2% addition of PE-MPs; 0.6: 0.6% addition of PE-MPs; and 1.0: 1.0% addition of PE-MPs. In figure (**A**), scale bar = 1 cm. In figure (**B**–**E**), error bars represent the standard deviation of the mean (*n* = 5). Different letters on the error bars indicate significant differences among treatments at each dpt (one-way analysis of variance—ANOVA; *p* < 0.05; Duncan’s multiple-range test). No letters on the error bars indicate there is no significant difference among treatments.

To evaluate the effects of different concentrations of PE-MPs on peanut responses to rhizobia, we documented phenotypic changes from 10 to 50 days post-treatment (dpt). Compared with the Control treatment, treatment with PE-MPs at concentrations of 0.2%, 0.6%, and 1.0% did not significantly affect peanut chlorophyll content (Figure 1A,B). However, these treatments obviously reduced peanut shoot dry weight, nodule number (with the exception of 1.0 treatment at 20 dpt), and nodule fresh weight after 20 dpt (Figure 1A,C–E). These results indicate that PE-MPs significantly inhibit the formation of peanut nodules. Notably, within the tested concentration range this inhibition does not exhibit a concentration-dependent effect. We speculate that the three concentrations tested in this study exceeded the threshold for influencing peanut nodulation. A finer gradient of concentrations, particularly at lower levels, is required to more precisely delineate this threshold.

### 2.2. Transcriptome Analysis

To further investigate the mechanisms of PE-MPs influencing peanut nodulation, we conducted transcriptomic profiling of peanut roots at 10 dpt and 20 dpt. RNA-Seq analysis generated an average of 46.37 million raw reads per library. After quality control, 45.63 million high-quality clean reads were retained per treatment. The sequencing data demonstrated excellent quality, with a Q30 score of 97.54% and an average GC content of 44.12%. Of these clean reads, 90.70–96.55% were successfully mapped to the *A. duranensis* reference genome, with unique mapping rates ranging from 77.61% to 83.45% (Table S1). These metrics confirm the high reliability and suitability of the data for subsequent in-depth analyses.

**Figure 2:**
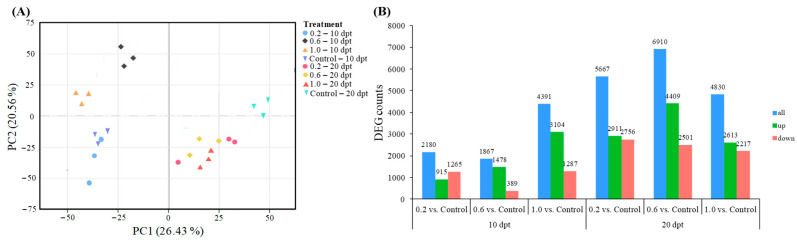
Principal component analysis (PCA) transcriptome analysis of peanut roots under the Control, 0.2, 0.6 and 1.0 treatments (*n* = 3) at 10 dpt and 20 dpt (**A**). Count of up- and down-regulated differentially expressed genes (DEGs) under the comparisons of 0.2 and Control, 0.6 and Control, and 1.0 and Control at 10 dpt and 20 dpt (**B**). In figure (**A**), principal coordinate axis 1 (PC1) accounts for the largest proportion of observed variance among samples, whereas axis 2 (PC2) represents the next most significant source of variation, independent of the patterns captured by PC1. Each point represents a sample, and the same color represents the same treatment. In figure (**B**), *p* ≤ 0.05, and letters on the column indicate the number of DEGs.

Principal component analysis (PCA) (Figure 2A) showed that samples within the same treatment exhibited highly similar transcriptomic profiles, with a combined variance explanation rate of 46.9% for the principal components. When projected along the PC1 axis, samples clearly segregated based on sampling time, forming distinct clusters for 10 dpt and 20 dpt. Along the PC2 axis, at 10 dpt, the 0.2 and Control treatments clustered together, while the 0.6 and 1.0 treatments formed a separate cluster. By 20 dpt, the 0.2, 0.6, and 1.0 treatments clustered together and were clearly separated from the Control. These results demonstrate distinct time-dependent transcriptional responses. At 10 dpt, the 0.6 and 1.0 treatments induced more substantial transcriptional alterations than the 0.2 or Control treatments. At 20 dpt, all PE-MP treatments triggered markedly more pronounced transcriptional changes compared to the Control. Statistical analysis of differentially expressed gene (DEG) counts further confirmed that treatment duration was a critical factor modulating peanut transcription. As shown in Figure 2B, the number of DEGs in the 0.2, 0.6, and 1.0 treatments at 20 dpt significantly exceeded those detected in the corresponding treatments at 10 dpt.

### 2.3. Gene Ontology (GO) Enrichment Analysis

**Figure 3:**
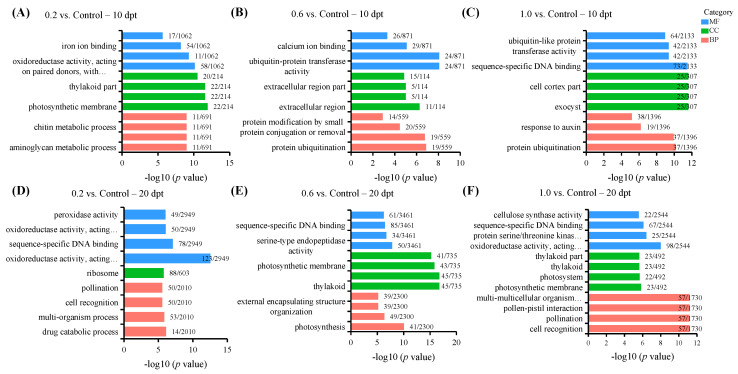
Gene Ontology (GO) enrichment analysis (*p* ≤ 0.05) of 0.2 compared with Control (**A**,**D**), 0.6 compared with Control (**B**,**E**), and 1.0 compared with Control (**C**,**F**) at 10 dpt and 20 dpt. The horizontal axis indicates the gene ratio, defined as the proportion of significantly enriched genes in a GO term relative to the total number of DEGs. The vertical axis represents significantly enriched GO terms, categorized into three standard ontologies: MF—molecular function; CC—cellular component; and BP—biological process. Letters on the column represent the gene ratio. Please refer to Table S2 for detailed information.

To capture the dynamic transcriptional shifts, we subsequently profiled metabolic pathways of PE-MP exposure. The top 12 enriched GO terms and associated molecular function (MF), cellular component (CC), and biological process (BP) category for each comparison group at 10 and 20 dpt are displayed in Figure 3 and Table S2. At 10 dpt, in 0.2 vs. Control, DEGs were primarily enriched in aminoglycan metabolic/catabolic processes (in the BP category), chitin catabolic process (BP, MF), oxidoreductase activity (MF), photosystem (CC), and iron ion binding (MF) (Figure 3A). In 0.6 vs. Control, DEGs were mainly associated with protein ubiquitination (BP, MF), defense response (BP), extracellular structure organization (CC), calcium ion binding (MF), and sequence-specific DNA binding (MF) (Figure 3B). In 1.0 vs. Control, DEGs were predominantly linked to protein ubiquitination (BP, MF), response to auxin (BP), cell cortex and cytoplasmic region (CC), sequence-specific DNA binding (MF), and calcium ion binding (MF) (Figure 3C). Collectively, at 10 dpt, all PE-MP treatments triggered peanut defense responses, such as chitin catabolic process and oxidoreductase activity in the 0.2 treatment, protein ubiquitination and defense response in the 0.6 treatment, as well as protein ubiquitination and response to auxin in the 1.0 treatment.

At 20 dpt, in 0.2 vs. Control, DEGs were primarily associated with defense responses (e.g., oxidoreductase and peroxidase activities in the MF category), signal transduction (e.g., cell recognition in the BP category and sequence-specific DNA binding in the MF category), drug catabolic process (BP), pollination (BP), and ribosomal components (CC) (Figure 3D). In 0.6 vs. Control, DEGs were mainly enriched in defense responses (e.g., cell wall organization/biogenesis and external encapsulating structure organization in the BP category), photosynthesis (e.g., photosynthesis in the BP category, photosystem in the CC category), growth regulation (e.g., serine-type endopeptidase activity and O-methyltransferase activity in the MF category), and signal transduction (e.g., sequence-specific DNA binding in the MF category) (Figure 3E). In 1.0 vs. Control, DEGs were predominantly linked to defense responses (e.g., oxidoreductase activity and cellulose synthase activity in the MF category), photosynthesis (e.g., photosystem and thylakoid in the CC category), signal transduction (e.g., cell recognition in the BP category and sequence-specific DNA binding in the MF category), and growth regulation (e.g., protein serine/threonine kinase activity in the MF category), as well as multi-multicellular organism process and pollination in the BP category (Figure 3F). In general, at 20 dpt, all PE-MP treatments consistently triggered peanut defense responses and signal transduction.

### 2.4. Kyoto Encyclopedia of Genes and Genomes (KEGG) Enrichment Analysis

**Figure 4:**
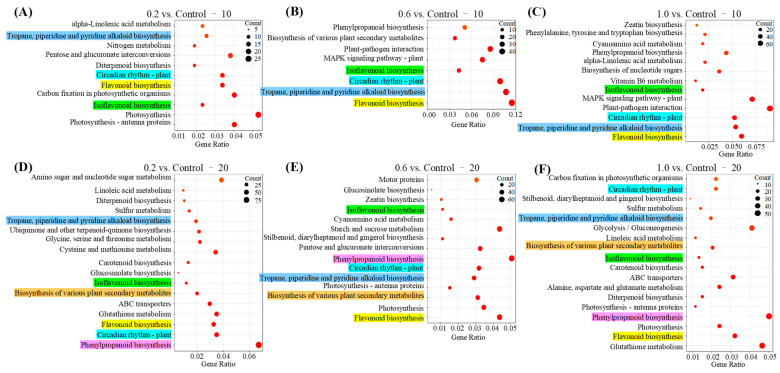
Kyoto Encyclopedia of Genes and Genomes (KEGG) enrichment analysis (*p* ≤ 0.05) of 0.2 compared with Control (**A**,**D**), 0.6 compared with Control (**B**,**E**), and 1.0 compared with Control (**C**,**F**) at 10 dpt and 20 dpt. The horizontal axis corresponds to the gene ratio, which reflects the enrichment degree of the pathway—values further to the right indicate a higher level of enrichment. The vertical axis displays the name of enriched KEGG pathways. The size of the bubble represents the number of DEGs annotated to a KEGG term. Pathways marked in the same color background are pathways shared by 0.2 vs. Control, 0.6 vs. Control, and 1.0 vs. Control comparisons. Among these, sky blue indicates Tropane, piperidine and pyridine alkaloid biosynthesis; cyan represents Circadian rhythm-plant; yellow denotes Flavonoid biosynthesis; green stands for Isoflavonoid biosynthesis; red marks Phenylpropanoid biosynthesis; and brown corresponds to the Biosynthesis of various plant secondary metabolites.

KEGG enriched pathways and associated details for each comparison group at 10 and 20 dpt are displayed in Figure 4 and Table S3. At 10 dpt, 0.2, 0.6 and 1.0 collectively influenced the pathways of Isoflavonoid biosynthesis (adu00943), Flavonoid biosynthesis (adu00941), Circadian rhythm-plant (adu04712), and Tropane, piperidine and pyridine alkaloid biosynthesis (adu00960) (Figure 4A–C). At 20 dpt, these three treatments further altered Isoflavonoid biosynthesis (adu00943), Flavonoid biosynthesis (adu00941), Circadian rhythm-plant (adu04712), Tropane, piperidine and pyridine alkaloid biosynthesis (adu00960), Biosynthesis of various plant secondary metabolites (adu00999), and Phenylpropanoid biosynthesis (adu00940) (Figure 4D–F). These results demonstrate that the biosynthetic pathways of isoflavonoids, flavonoids, tropane/piperidine/pyridine alkaloids, and circadian rhythm were significantly disrupted at 10 dpt of PE-MPs exposure, with effects persisting into 20 dpt. Additionally, the Phenylpropanoid biosynthesis pathway and Biosynthesis of various plant secondary metabolites pathway were specifically altered at 20 dpt. Collectively, these shared metabolic perturbations across all three PE-MP treatments likely contribute to the significant reduction in peanut nodule numbers. As these transcriptomic results represent a preliminary overview of how PE-MPs influence the symbiosis of peanut and rhizobia, further functional validation—including metabolite quantification, enzyme activity assays, and direct observation of rhizobial infection—is essential to establish causal links between the observed gene expression changes and impaired nodulation.

### 2.5. DEG Function Analysis

**Figure 5:**
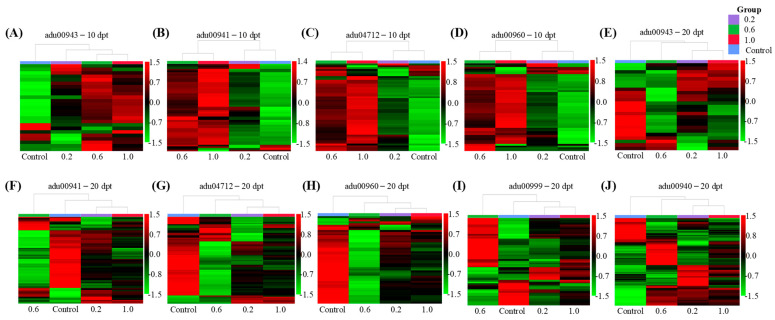
In the Control, 0.2, 0.6, and 1.0 treatments, heat maps of DEGs in the pathways of Isoflavonoid biosynthesis (adu00943) (**A**), Flavonoid biosynthesis (adu00941) (**B**), Circadian rhythm-plant (adu04712) (**C**), and Tropane, piperidine and pyridine alkaloid biosynthesis (adu00960) (**D**) at 10 dpt; and heat maps of DEGs in the pathways of Isoflavonoid biosynthesis (adu00943) (**E**), Flavonoid biosynthesis (adu00941) (**F**), Circadian rhythm-plant (adu04712) (**G**), Tropane, piperidine and pyridine alkaloid biosynthesis (adu00960) (**H**), Biosynthesis of various plant secondary metabolites (adu00999) (**I**), and Phenylpropanoid biosynthesis (adu00940) (**J**) at 20 dpt. Red, black and green separately represent the up-regulated, unchanged and down-regulated DEGs.

DEGs associated with KEGG pathways jointly affected by the 0.2, 0.6 and 1.0 treatments were analyzed using clustering heat maps. At 10 dpt, the distribution of DEGs in the Isoflavonoid biosynthesis pathway clearly separated the 0.2, 0.6 and 1.0 treatments from the Control (Figure 5A). Functional analysis results showed that five DEGs enriched by 0.2, 0.6 and 1.0 treatments increased the synthesis of key isoflavonoids, including 2′-Hydroformononetin, Medicarpin, Sophorol, 2′-Hydroxygenistein, and 2′-Hydroxybiochanin A (Figure S1A, Table S4). These compounds serve as critical signaling molecules in peanut, inducing rhizobia to synthesis nodulation (Nod) factors and initiating nodule formation [9]. The distribution of DEGs in the Flavonoid biosynthesis pathway, Circadian rhythm-plant, and Tropane, piperidine and pyridine alkaloid biosynthesis pathway showed distinct clustering, separating the 0.6 and 1.0 treatments from the 0.2 and Control treatments (Figure 5B–D). Functional analysis revealed that these three PE-MP treatments collectively enriched 10 DEGs associated with chalcone synthase (CHS) synthesis (Table S4). In the Flavonoid biosynthesis pathway, CHS catalyzes the production of nodulation regulatory factors and antioxidant precursors such as pinocembrin chalcone, phloretin, naringenin chalcone, eriodictyol chalcone, and homoeriodictyol chalcone (Figure S1B) [14,15]. Within the Circadian rhythm pathway, CHS contributes to UV-B radiation protection by facilitating the synthesis of protective flavonoids (Figure S1C) [16]. Simultaneously, in the Tropane, piperidine, and pyridine alkaloid biosynthesis pathway, CHS catalyzes the formation of 4-(1-methyl-2-pyrrolidinyl)-3-oxobutanoic acid, a key biosynthetic precursor for tropine (Figure S1D). Tropine therefore contributes to plant defense against microbial invasion through its well-characterized antimicrobial and defense-modulating activities [17].

At 20 dpt, the distribution of DEGs associated with the Isoflavonoid biosynthesis pathway, Circadian rhythm-plant, Tropane, piperidine and pyridine alkaloid biosynthesis pathway, and Phenylpropanoid biosynthesis pathway in the 0.2, 0.6 and 1.0 treatments exhibited clear separation from the Control treatment, indicating distinct transcriptional reprogramming induced by PE-MPs (Figure 5E,G,H,J). Functional analysis results showed that four DEGs in the Isoflavonoid biosynthesis pathway under the 0.2, 0.6 and 1.0 treatments significantly suppressed the synthesis of key isoflavonoids, including 2′-Hydroformononetin, 2′-Hydroxygenistein, 2′-Hydroxybiochanin A, 2′-Hydroxyformononetin, and 2′,7-Dihydroxy-4′,5′-methylenedioxy isoflavone (Figure S2A, Table S5). In Circadian rhythm-plant, reduced expression of 13 CHS-encoding DEGs damaged UV-B protective capability (Figure S2C, Table S5). In the Tropane, piperidine and pyridine alkaloid biosynthesis pathway, down-regulation of 13 CHS-encoding DEGs reduced the synthesis of tropine precursor 4-(1-methyl-2-pyrrolidinyl)-3-oxobutanoic acid (Figure S2D, Table S5). In the Phenylpropanoid biosynthesis pathway, 32 DEGs promoted the synthesis of key lignin polymers, including p-Hydroxyphenyl lignin (H-lignin), Guaiacyl lignin (G-lignin), 5-Hydroxy-guaiacyl lignin (5H-lignin), and Syringyl lignin (S-lignin) (Figure S2F, Table S5). The deposition of lignin enhances plant resistance to rhizobial infection through cell wall thickening [18]. Conversely, the distribution of DEGs in the Flavonoid biosynthesis pathway and Biosynthesis of various plant secondary metabolites pathway in the Control, 0.2 and 2.0 treatments were separated from those in the 0.6 treatment (Figure 5F,I). Functional analysis further demonstrated that in the Flavonoid biosynthesis pathway, 22 DEGs encoding key enzymes (e.g., CHS and caffeoyl-CoA O-methyltransferase) enhanced the metabolic flux toward feruloyl-CoA accumulation, while suppressing the synthesis of antioxidant precursors such as pinocembrin chalcone, phloretin, naringenin chalcone, eriodictyol chalcone, homoeriodictyol chalcone, and isoliquiritigenin (Figure S2B, Table S5). Feruloyl-CoA serves as a critical precursor for diverse flavonoids and lignin. In the Biosynthesis of various plant secondary metabolites pathway, 12 DEGs promoted the accumulation of key intermediates, including S-adenosyl-L-methionine (SAM), 2-coumarinate, and scopoline (Figure S2E, Table S5). These compounds are known to contribute to plant defense mechanisms against microorganisms [19,20].

### 2.6. Protein–Protein Interaction Analysis

**Figure 6:**
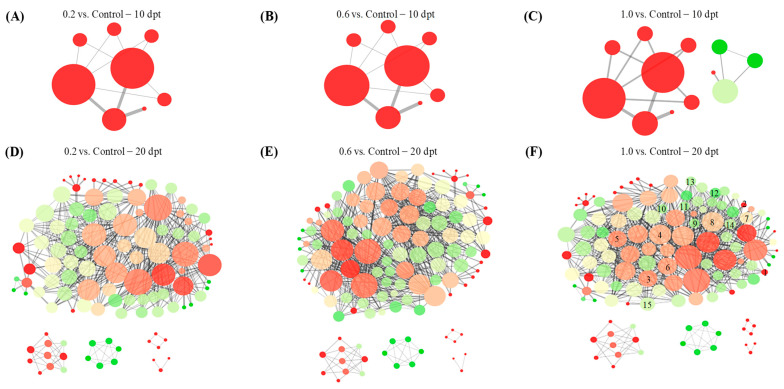
Protein–protein interaction (PPI) network maps regulated by 0.2 (**A**,**D**), 0.6 (**B**,**E**) and 1.0 (**C**,**F**) treatments at 10 dpt and 20 dpt. Each node represents a protein, and each connection line represents the interaction between the connected proteins. The size and color depth of nodes represent protein expression level (positive correlation). The thickness and color of junction lines represent the correlation between proteins (positive correlation). Red, yellow and green separately represents the up-regulated, unchanged and down-regulated DEGs. In figure (**F**), nodes 1–15 represent proteins that are absent in the 0.2 treatment compared to the 1.0 treatment. Similarly, node 15 corresponds to a protein that is missing in the 0.6 treatment relative to the 1.0 treatment.

Protein–protein interaction (PPI) network analysis revealed structurally similar networks among all the three treatments. At 10 dpt, the PPI networks for the 0.2 and 0.6 treatments were identical. The 1.0 treatment shared the same core PPI architecture but contained four additional peripheral interacting proteins absent in the lower-concentration treatments (Figure 6A–C). At 20 dpt, the complexity of the PPI networks increased significantly, comprising 111, 126, and 127 interacting proteins for the 0.2, 0.6, and 1.0 treatments, respectively. Despite this increased complexity, the overall network architectures and core protein components remained highly conserved among these treatments (Figure 6D–F). These results indicate that the transcriptional influence of PE-MPs on peanut is minimal at 10 dpt but becomes more pronounced and complex at 20 dpt. Furthermore, compared to the 1.0 treatment, the 0.2 treatment lacked proteins represented by nodes 1–15, while the 0.6 treatment specifically lacked the protein corresponding to node 15 (Figure 6F). These findings suggest that within the tested concentration range (0.2–1.0%), variations in PE-MP concentration exert limited effects on the overall transcriptional response in peanut.

## 3. Discussion

While numerous studies have investigated the potential toxicity of PE-MPs on soil properties, microbial communities, and crop physiology [21,22,23], their effects on rhizobial symbiosis with leguminous crops remain largely unexplored. Our study addressed this critical knowledge gap by systematically evaluating how PE-MPs modulated peanut nodulation efficiency. The results demonstrated a strong time-dependent response, wherein the impact of PE-MPs on peanut transcriptomes was significantly less pronounced at 10 dpt than at 20 dpt. In contrast, the effect of concentration variation was relatively minor. Notably, all tested PE-MP concentrations (0.2%, 0.6%, and 10%) significantly reduced nodule numbers compared to the Control, though no statistically significant differences were observed among these PE-MP treatments. Collectively, these findings reveal a previously overlooked aspect of MP phytotoxicity—specifically, its capacity to disrupt the balance of legume responses to beneficial microorganisms.

From 10 to 20 dpt, exposure to 0.2%, 0.6%, and 1.0% PE-MPs consistently perturbed four key metabolic pathways in peanut: Isoflavonoid biosynthesis, Flavonoid biosynthesis, Circadian rhythm-plant, and Tropane, piperidine, pyridine alkaloid biosynthesis. These pathways represent core metabolic responses to PE-MP stress, though the expression levels of associated DEGs differed significantly between 10 dpt and 20 dpt. In the Isoflavonoid biosynthesis pathway, the synthesis of hydroxylated isoflavones (e.g., 2′-Hydroxygenistein, 2′-Hydroxybiochanin A, and 2′-Hydroxyformononetin) was significantly up-regulated at 10 dpt but markedly down-regulated by 20 dpt. As these compounds regulate rhizobial Nod factor synthesis and nodulation processes [9], their decline correlates with reduced nodule numbers. In the Flavonoid biosynthesis pathway, chalcone-type flavonoids (e.g., pinocembrin chalcone, phloretin, naringenin chalcone, eriodictyol chalcone, homoeriodictyol chalcone, and isoliquiritigenin) showed similar temporal dynamics—up-regulated at 10 dpt and down-regulated at 20 dpt. These flavonoids play a crucial role in mediating nodulation and antioxidant activity in leguminous plants [14,15]. The observed reduction in their synthesis likely compromises nodulation capabilities in peanut. In the Circadian rhythm pathway, the UV-B radiation protection capacity increased at 10 dpt but decreased at 20 dpt. Circadian disruption or diminished UV-B defense capacity impairs temporal coordination of defense responses [24], leading to reactive oxygen species (ROS) accumulation and programmed cell death (PCD) [25]. This defensive response compromises the reproduction of rhizobia within peanut root cells. In the Tropane, piperidine, pyridine alkaloid biosynthesis pathway, synthesis of the tropine precursor 4-(1-methyl-2-pyrrolidinyl)-3-oxobutanoic acid was up-regulated at 10 dpt but down-regulated at 20 dpt. This precursor supports defense via antimicrobial activity and ROS scavenging [17]. The reduction in its quantity may be responsible for the low level of nodule formation in peanut. In summary, the coordinated down-regulation of hydroxylated isoflavones, chalcone-type flavonoids, and tropine precursors, as well as an unbalanced antioxidant activity system, collectively contributed to the reduction in peanut nodulation capacity. It is worth noting that the transcriptomic changes observed here merely reflect the response of the peanut–rhizobia symbiosis to PE-MPs under the specific conditions tested. Therefore, subsequent experiments—such as metabolite quantitation or physiological validation—are required to substantiate these interpretations.

At 20 dpt, the 0.2, 0.6, and 1.0 treatments additionally perturbed the Biosynthesis of various plant secondary metabolites and Phenylpropanoid biosynthesis pathways. In the Biosynthesis of various plant secondary metabolites pathway, significant accumulation of SAM, 2-coumarinate, and scopolin was observed. Specifically, SAM functions as a key methyl donor in lignin and ethylene biosynthesis, both of which are critical for defense responses while simultaneously suppressing rhizobial infection and nodule formation [26,27]. 2-coumarinate serves as a metabolic gateway to coumarin-derived antimicrobial compounds [20]. Scopolin enhances microbial defense as a phytoalexin or antimicrobial precursor [19]. Concurrently, the Phenylpropanoid biosynthesis pathway exhibited extensive accumulation of diverse lignin forms (H-, G-, 5H-, and S-lignin). Lignin deposition reinforces physical barriers by fortifying cell walls, restricting rhizobial infection, thereby inhibiting nodules formation [18,28]. Overall, the thickening of the cell wall, coupled with the extensive synthesis of various secondary metabolites associated with antimicrobial properties, collectively contributes to preventing peanut from infection by rhizobia.

Based on previously reported concentrations of PE-MPs in agricultural soils, typically ranging from 0.03% to 6.70% [29], this study established three PE-MP application levels (0.2%, 0.6%, 1.0%) to evaluate their ecological impacts on peanut under low-, moderate-, and high-pollution scenarios. Results demonstrated that even at the 0.2% level, peanut nodule numbers decreased significantly, indicating that current environmental PE-MP concentrations may already exert measurable adverse effects on peanut nodulation. Furthermore, it was observed that while PE-MPs at concentrations of 0.2%, 0.6%, and 1.0% significantly inhibited peanut nodulation, the reduction in nodule numbers did not exhibit a proportional dose–response relationship with increasing PE-MP concentrations. This phenomenon may be attributed to the substantial impact of low PE-MP concentration (0.2%) on key metabolic pathways, and 0.2% concentration may already exceed the threshold for exerting a significant effect on peanut nodulation. Therefore, to determine whether a threshold concentration exists for PE-MP inhibition of peanut nodulation, subsequent experiments should examine the effects of a wider range of lower concentrations. Furthermore, the dose-independent mechanisms suggested by our findings require more direct experimental validation. This concentration-independent effect of MPs on plants is consistent with the findings of Zhang et al. (2023) [30], who observed a non-significant correlation between the concentration of polystyrene microplastics (PS-MPs) and multiple rice germination parameters, including germination percentage, germination vigor, and germination index. A fundamental limitation of this study lies in its short-term, controlled pot design, which cannot replicate the dynamic, multi-stress conditions of real agricultural soils. The critical question—how chronic exposure to weathered PE-MPs, interacting with fluctuating environmental factors and the native microbiome, affects peanut nodulation over multiple growing seasons—remains unanswered. Addressing this gap is not merely a matter of confirming our findings, but of fundamentally re-evaluating the ecological threshold and long-term risks of MP pollution under realistic field conditions.

When analyzed jointly with the findings of Wu et al. (2024) [9], we observed that the adverse effects of MPs on peanut–rhizobia symbiosis are conserved across MP types. PE, polyvinyl chloride (PVC), and polybutylene adipate (PBAT) MPs consistently suppressed peanut nodulation capacity and disrupted core metabolic pathways related to defense and symbiosis, including Flavonoid/Isoflavonoid biosynthesis, Circadian rhythm, Tropane, piperidine, and pyridine alkaloid biosynthesis. However, the specific molecular targets within these pathways exhibited polymer-dependent variations. For instance, PE-MPs predominantly altered the synthesis of chalcone flavonoids (e.g., pinocembrin chalcone, naringenin chalcone), hydroxylated isoflavone (e.g., 2′-Hydroformononetin, 2′-Hydroxygenistein), and the tropine precursor 4-(1-methyl-2-pyrrolidinyl)-3-oxobutanoic acid, while concurrently compromising UV-B protection mechanisms. PVC-/PBAT-MPs primarily affected the accumulation of prunetin and naringenin, altered tropine biosynthesis, and disrupted light signal transduction components (e.g., phytochrome A activity). We infer that the observed differences may result from the distinct experimental conditions: PE-MPs were applied in field-based pot trials, whereas PVC-/PBAT-MPs were tested under greenhouse vermiculite cultivation. These contrasting cultivation systems likely led to disparities in rhizobial community structure of peanut roots, variations in the physicochemical properties of the growth substrate, and differing climatic conditions, ultimately causing variations in the expression of the aforementioned metabolic genes.

The disruption of the peanut–rhizobium symbiosis by MPs reduces the nitrogen supply derived from biological nitrogen fixation in peanut, thereby increasing reliance on chemical nitrogen fertilizers. This shift in nutrient sourcing may trigger a cascade of ecological consequences, including soil compaction, acidification, and microbial community imbalances resulting from long-term chemical fertilizer application [31]. Consequently, although MP pollution may be quantitatively inconspicuous, it can exert a progressively amplified effect along the chain of rhizobium–legume–nitrogen fertilizer–soil environment, leading to serious cascading impacts on soil ecosystems. In summary, our findings demonstrate that low, medium, and high environmental concentrations of PE-MPs inhibit peanut nodulation by disrupting key metabolic pathways. These results provide critical mechanistic insights and empirical data for evaluating the effects of PE-MPs under real-world peanut cultivation conditions, while also underscoring the need for ongoing monitoring and comprehensive risk assessment of MPs as an emerging agricultural pollutant with potentially far-reaching implications.

It is worth noting that in this study, only one diploid parent—*A. duranensis*, which contributes the A sub-genome—was used as the reference genome. Since the cultivated peanut is a tetraploid (AABB) containing both A and B sub-genomes, this approach may lead to the loss of sequence information from the B sub-genome, potentially resulting in a reduced read alignment rate and biased expression quantification. Therefore, the findings of this study reflect only gene expression associated with the A sub-genome and do not account for genes specifically expressed from the B sub-genome.

## 4. Materials and Methods

### 4.1. Experimental Design

PE-MPs used in this study were purchased from Jing Tian Plastic Raw Materials Co., Ltd., Dongguan, China. Scanning electron microscopy (SEM) images and Fourier transform infrared spectra of PE-MPs are shown in Figure S3. SEM images showed that PE-MPs were relatively smooth, with few obvious folds or pores. The MPs overall appeared as spherical particles, and the actual diameter of PE-MPs was 1–50 μm and the density was 0.91 g/cm^3^. The experimental soil was collected from the top layer (0–20 cm depth) of a peanut plantation in Laixi City, Shandong Province, China (E 120°29′, N 36°48′). This site has experienced continuous peanut cultivation for 6 years. According to Chinese Soil Taxonomy [32], the soil type was brown soil and the texture was clay loam soil. Physicochemical properties of the soil before planting peanut are provided in Table S6.

The peanut variety was BS1016 (cultivated *Arachis hypogaea* L. variety), which was preserved by Shandong Peanut Research Institute. Before planting, peanut seeds were germinated by soaking in deionized water for 3–4 h, followed by wrapping in moist gauze and incubating in darkness at 28 °C for 2 days. Peanut seedlings with roots approximately 1 cm in length were soaked for 5 min in a rhizobial suspension (*Bradyrhizobium zhanjiangense* CCBAU 51778, with a concentration of 1 × 10^8^ cfu/mL) before planting. PE-MPs were incorporated into the soil, which was then transferred to the pot. Each pot was filled with 2.5 kg mixed soil and planted with one peanut seedling. Before this, a layer of sand and six layers of cotton gauze were positioned at the bottom of the pot, which could prevent PE-MPs from being carried away with the excess water. To simulate the real field environment, we buried the pots in a peanut field, with the top of the pot only 5 cm above the soil surface. Peanut seedlings were cultivated in these pots and watered when needed. To minimize positional effects in the field, all pots were initially arranged in a randomized design and were subsequently picked up and re-randomized every 10 days. A study has indicated that the concentration of MPs in soil may range from 0.03% to 6.70% [29]. To account for the potential accumulation of PE-MPs from field runoff and agglomerated waste film, we added low (0.2%, *w*/*w*, MPs weight/dry soil weight), medium (0.6%) and high (1.0%) concentrations of PE-MPs into the soil. To prepare the soil–MPs mixture, pre-weighed PE-MP powder was incorporated into the soil in batches. The mixture was then stirred thoroughly with a shovel to ensure a visually homogeneous distribution. Four treatments were set up: (1) Control, no PE-MP addition; (2) 0.2, 0.2% (*w*/*w*) PE-MP addition; (3) 0.6, 0.6% (*w*/*w*) PE-MP addition; (4) 1.0, 1.0% (*w*/*w*) PE-MPs addition. For each treatment in this experiment, 31 potted plants were established (1 plant/pot).

### 4.2. Phenotypic Assessment of Peanut Plants

The assessment of peanut phenotypic traits comprised two components: (1) imaging of leaf and nodule samples collected at 40 dpt; (2) quantitative measurements of chlorophyll content (SPAD value), shoot dry weight, nodule number, and nodule fresh weight conducted at 10, 20, 30, 40, and 50 dpt, following the protocol described by Wu et al. (2024) [9]. For each treatment and time point, five independent plants were sampled (*n* = 5).

Statistical analyses were performed using SPSS version 20.0 (IBM, Armonk, NY, USA). All datasets were first tested for conformity to a normal distribution using the Shapiro–Wilk test and for homogeneity of variances using Levene’s test. The results indicated that the data satisfied the prerequisites for parametric analysis (*p* > 0.05 for both tests). Subsequently, one-way analysis of variance (ANOVA) was applied to determine whether significant differences existed among treatments, followed by Duncan’s multiple range test for post hoc comparisons, with statistical significance defined at *p* < 0.05. Data throughout the study are reported as the mean ± standard deviation (SD), where *n* = 5 represents five biological replicates per treatment per time point.

### 4.3. Transcriptomic Analysis of Peanut Roots

Root samples were collected at 10 and 20 dpt, corresponding to the initial emergence and full maturation of peanut nodules, respectively. For each time point and treatment, samples were taken from three independent plants (*n* = 3). The collected samples were immediately snap-frozen in liquid nitrogen and stored for subsequent transcriptomic analysis.

For transcriptome analysis, total RNA of each sample was extracted using an RNAprep Pure Plant Kit (Tiangen, Beijing, China) following the established protocols [9], with RNA quality (including purity, concentration, and integrity) verified via an Agilent 2100 bioanalyzer (Agilent Technologies, Santa Clara, CA, USA) and NanoDrop spectrophotometer (Thermo Fisher Scientific, Wilmington, DE, USA). mRNA with a polyA tail was enriched using Oligo (dT) magnetic beads and fragmented by the NEB Fragmentation Buffer. The first-strand cDNA was synthesized by M-MuLV reverse transcriptase using fragmented mRNA as the template and random oligonucleotide as primers. RNase H was used to degrade the RNA strand, and a second cDNA strand was synthesized using dNTP as a substrate in the DNA polymerase I system. Subsequently, the double-stranded cDNA was purified, followed by end repair, A-tailing, and ligation to a sequencing adapter. cDNA fragments of 250–300 bp were size-selected using AMPure XP beads, followed by PCR amplification. The resulting PCR products were purified using AMPure XP beads to construct the final sequencing library. The library was sequenced on the Illumina NovaSeq 6000 platform (Illumina, San Diego, CA, USA) by Nuohe Zhiyuan Bio-information Technology Co., Ltd., (Beijing, China). Peanut raw transcriptome data have been deposited in the National Center for Biotechnology Information (NCBI) Sequence Read Archive (SRA) database under the accession no. of PRJNA1253781.

Raw sequencing data was filtered, error rate evaluated, and GC content distribution analyzed to generate high-quality clean reads. Clean reads were then aligned to peanut reference genome (PeanutBase: https://www.peanutbase.org) using HISAT2 (v2.0.5) [33], and the information of these reads was obtained. Expression levels of identified genes were quantified using the fragments per kilobase per million mapped reads (FPKM) value. DESeq2 software (v1.20.0) was used for differential expression analysis between two treatments [34]. DEGs were identified using the criteria of |log2 (fold change)| ≥ 0 and adjusted *p*-value (*p* adj) ≤ 0.05 in different comparisons. Functional annotation of DEGs was performed using the ClusterProfiler (v3.8.1) [35] for both GO and KEGG analyses. The enriched GO terms and KEGG pathways for DEGs were identified using a significance threshold of *p* adj ≤ 0.05. GO enrichment analysis of DEGs was conducted to elucidate their functional roles across three main categories: MF, CC, and BP. FPKM values of DEGs were used for heat map cluster analysis. The interaction relationships in the STRING protein interaction database were applied to analyze the PPI network of DEGs. The reference species was *Arachis hypogaea*. And the Cytoscape (v.3.3.0) software was used to draw PPI network map.

## 5. Conclusions

This study provides comprehensive insights into the effects of PE-MPs on peanut responses to beneficial microorganisms. Concentrations of 0.2%, 0.6%, and 1.0% PE-MPs significantly suppressed peanut nodulation through reducing nodulation-related hydroxylated isoflavones and chalcone-type flavonoids, increasing the thickness of the cell wall, producing antimicrobial substances, and reducing antioxidant activity. This work not only reveals, for the first time at the transcriptomic level, the mechanisms by which PE-MPs affect legume nodulation, but also identifies critical directions for future inquiry. Subsequent studies should integrate multi-dimensional functional validation—including metabolic, physiological, and molecular approaches—to establish causality between these transcriptional changes and nodulation phenotypes, thereby offering a more comprehensive and forward-looking scientific perspective to the field. In general, this study provides new insights into the ecological risks of MP pollution in agricultural systems, addresses a critical gap in MP ecotoxicology, and offer a scientific basis for developing sustainable management strategies to mitigate MP contamination in farmland.

## Data Availability

The data presented in this study are available on request from the corresponding author.

## Supplementary Materials

The following supporting information can be downloaded at: https://www.mdpi.com/article/10.3390/plants15060915/s1, Figure S1: At 10 dpt, the Isoflavonoid biosynthesis pathway (adu00943) (**A**), Flavonoid biosynthesis pathway (adu00941) (**B**), Circadian rhythm-plant (adu04712) (**C**), and Tropane, piperidine and pyridine alkaloid biosynthesis pathway (adu00960) (**D**) were regulated by 0.2, 0.6 and 1.0 treatments. The up- or down-regulated DEGs were represented by “+” or “−”. Red fonts and red ellipses represented the DEGs and circled products were regulated by 0.2, 0.6 and 1.0 treatments. Figure S2: At 20 dpt, the Isoflavonoid biosynthesis pathway (adu00943) (**A**), Flavonoid biosynthesis pathway (adu00941) (**B**), Circadian rhythm-plant (adu04712) (**C**), Tropane, piperidine and pyridine alkaloid biosynthesis pathway (adu00960) (**D**), Biosynthesis of various plant secondary metabolites (adu00999) (**E**), and Phenylpropanoid biosynthesis pathway (adu00940) (**F**) were regulated by 0.2, 0.6 and 1.0 treatments. The up- or down-regulated DEGs were represented by “+” or “−”, respectively. Red fonts and red ellipses represented the DEGs and circled products were regulated by 0.2, 0.6 and 1.0 treatments. Figure S3: Scanning electron microscopy image (**A**) and Fourier transform infrared spectrum (**B**) of PE-MPs. Table S1: Quality of sequencing data. Table S2: Statistical information of GO-annotated DEGs. Table S3: Statistical information of KEGG-annotated DEGs. Table S4: Gene expression levels of KEGG-annotated DEGs of 0.2, 0.6, and 1.0 treatments compared with the Control at 10 dpt. Table S5: Gene expression levels of KEGG-annotated DEGs of 0.2, 0.6, and 1.0 treatments compared with the Control at 20 dpt. Table S6: Soil physicochemical properties.
