# Supplementary material for: Polyethylene Microplastics Inhibit Peanut Nodulation via Metabolic and Transcriptional Pathways
Source: Plants (Basel). 2026 Mar 16;15(6):915. doi: 10.3390/plants15060915 (PMC13030837; doi:10.3390/plants15060915)
Supplement: Supplementary file 1 [file plants-15-00915-s001.zip › Supplementary figures.pdf]

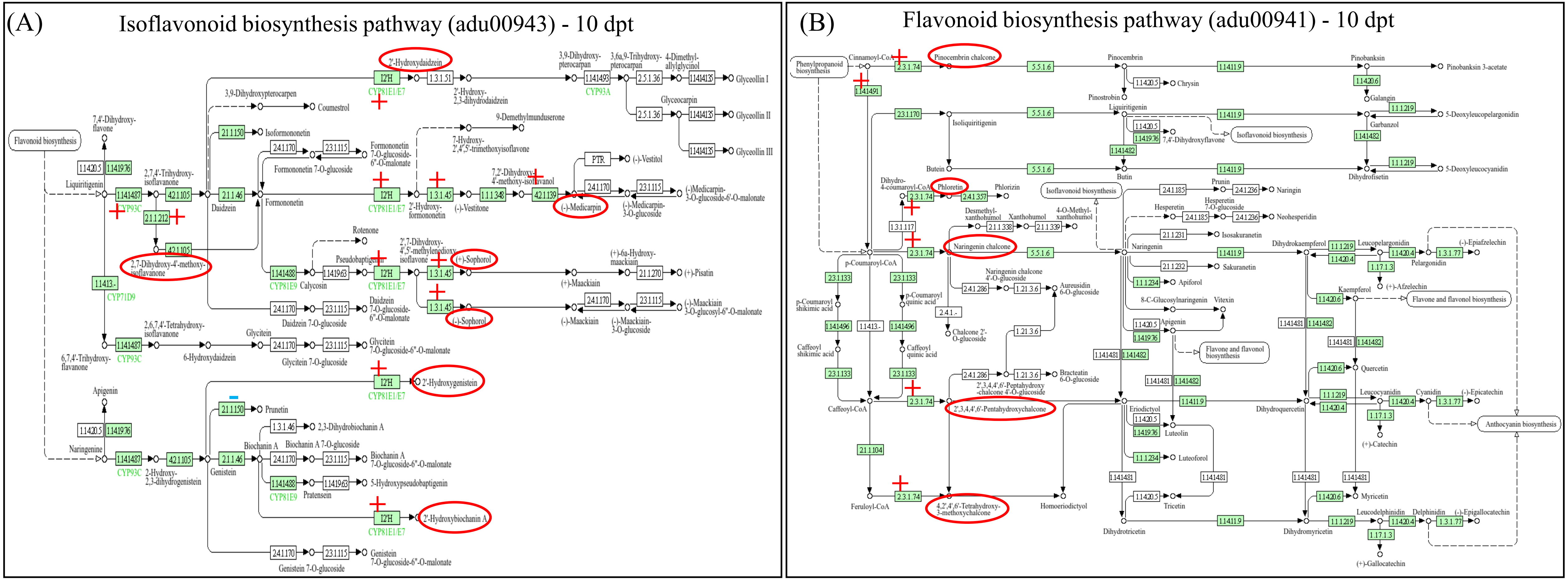

Figure S1. At 10 dpt, Isoflavonoid biosynthesis pathway (adu00943) (A), Flavonoid biosynthesis pathway (adu00941) (B), Circadian rhythm - plant (adu04712) (C), and Tropane, piperidine and pyridine alkaloid biosynthesis pathway (adu00960) (D) were regulated by 0.2, 0.6 and 1.0 treatments. The up- or down- regulated DEGs were represented by "+" or "-". Red fonts and red ellipses represented the DEGs and circled products were regulated by 0.2, 0.6 and 1.0 treatments.

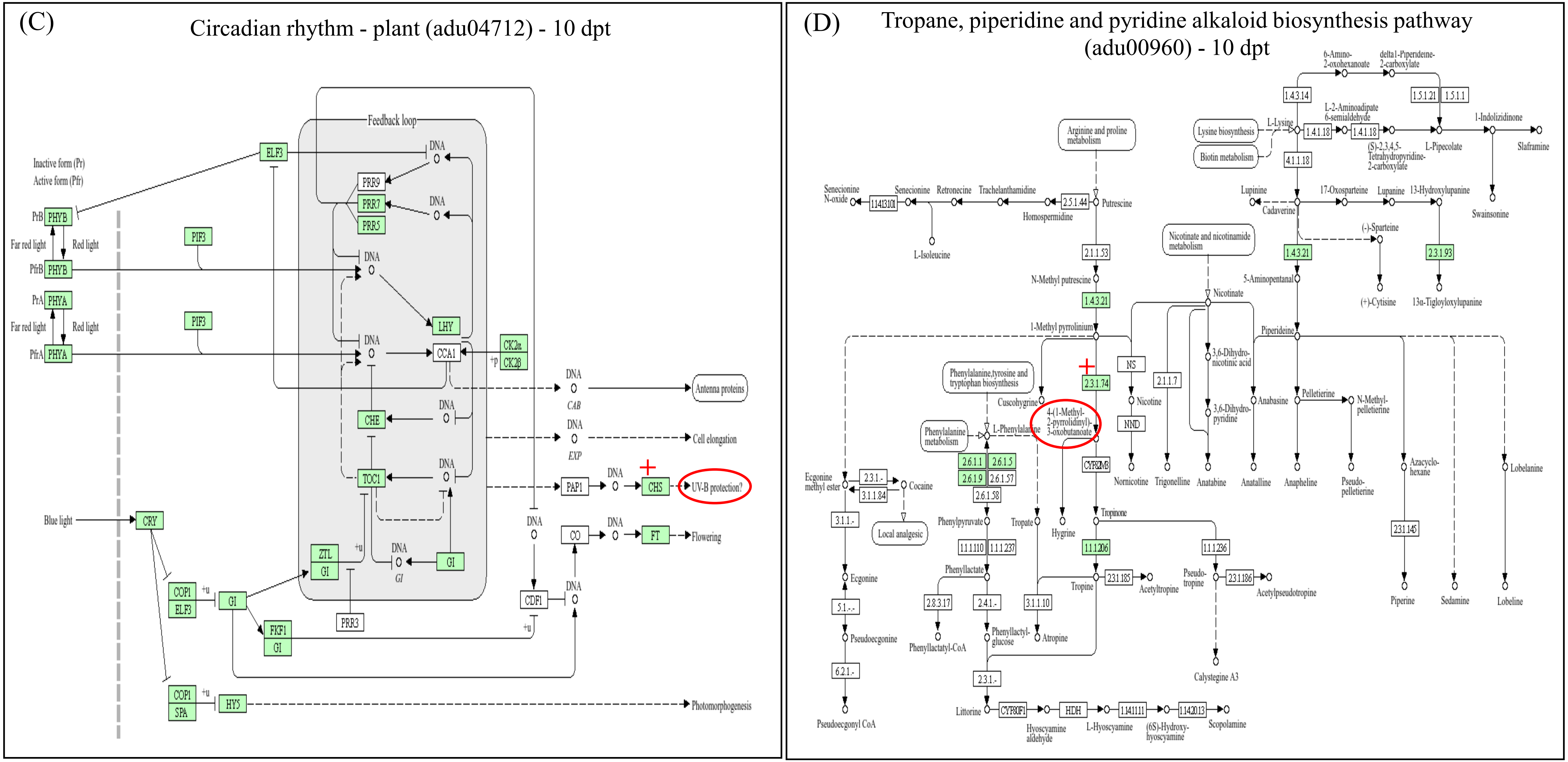
(C)
Circadian rhythm - plant (adu04712) - 10 dpt
Feedback loop
Inactive form (Pr)
Active form (Pfr)
PrB PHYB
PfrB PHYB
PrA PHYA
PfrA PHYA
Far red light
Red light
PIF3
ELF3
PRR9
PRR7
PRR5
DNA
LHY
CCA1
CK2α
CK2β
CHE
TOC1
ZTL
GI
FKF1
PRR3
CDF1
CO
FT
PAP1
CHS
CAB
EXP
Antenna proteins
Cell elongation
UV-B protection?
Flowering
Photomorphogenesis
Blue light
CRY
COP1
SPA
HY5
(D)
Tropane, piperidine and pyridine alkaloid biosynthesis pathway
(adu00960) - 10 dpt
Arginine and proline metabolism
Lysine biosynthesis
Biotin metabolism
Nicotinate and nicotinamide metabolism
Phenylalanine,tyrosine and tryptophan biosynthesis
Phenylalanine metabolism
Putrescine
N-Methyl putrescine
1-Methyl pyrrolinium
4-(1-Methyl-2-pyrrolidinyl)-3-oxobutanoate
Cuscohygrine
Hygrine
Tropinone
Tropine
Pseudo-tropine
Acetyltropine
Acetylpseudotropine
Calystegine A3
L-Phenylalanine
Phenylpyruvate
Phenyllactate
Phenyllactyl-CoA
Phenyllactyl-glucose
Littorine
Hyoscyamine aldehyde
L-Hyoscyamine
(6S)-Hydroxy-hyoscyamine
Scopolamine
Tropate
Atropine
Ecgonine methyl ester
Cocaine
Local analgesic
Ecgonine
Pseudoecgonine
Pseudoecgonyl CoA
Senecionine N-oxide
Senecionine
Retronecine
Trachelanthamidine
Homospermidine
L-Isoleucine
Nicotinate
3,6-Dihydro-nicotinic acid
3,6-Dihydro-pyridine
NS
NND
Nicotine
Nornicotine
Trigonelline
Anatabine
Anatalline
Anabasine
Anapheline
Pelletierine
N-Methyl-pelletierine
Pseudo-pelletierine
Azacyclo-hexane
Piperidine
Piperine
Sedamine
Lobelanine
Lobeline
L-Lysine
Cadaverine
5-Aminopentanal
6-Amino-2-oxohexanoate
delta1-Piperideine-2-carboxylate
L-2-Aminoadipate 6-semialdehyde
(S)-2,3,4,5-Tetrahydropyridine-2-carboxylate
L-Pipecolate
1-Indolizidinone
Slaframine
Swainsonine
Lupinine
17-Oxosparteine
Lupanine
13-Hydroxylupanine
(-)-Sparteine
(+)-Cytisine
13α-Tigloyloxylupanine
CYP80F1
HDH
2.3.1.74
1.4.3.21
2.1.1.53
2.5.1.44
2.6.1.1
2.6.1.5
2.6.1.9
2.6.1.57
2.6.1.58
1.1.1.206
1.1.1.236
2.3.1.185
2.3.1.186
3.1.1.10
2.8.3.17
2.4.1.-
2.3.1.-
3.1.1.84
3.1.1.-
5.1.-.-
6.2.1.-
1.4.3.14
1.5.1.21
1.5.1.1
1.4.1.18
4.1.1.18
2.3.1.93
2.3.1.145
2.1.1.7

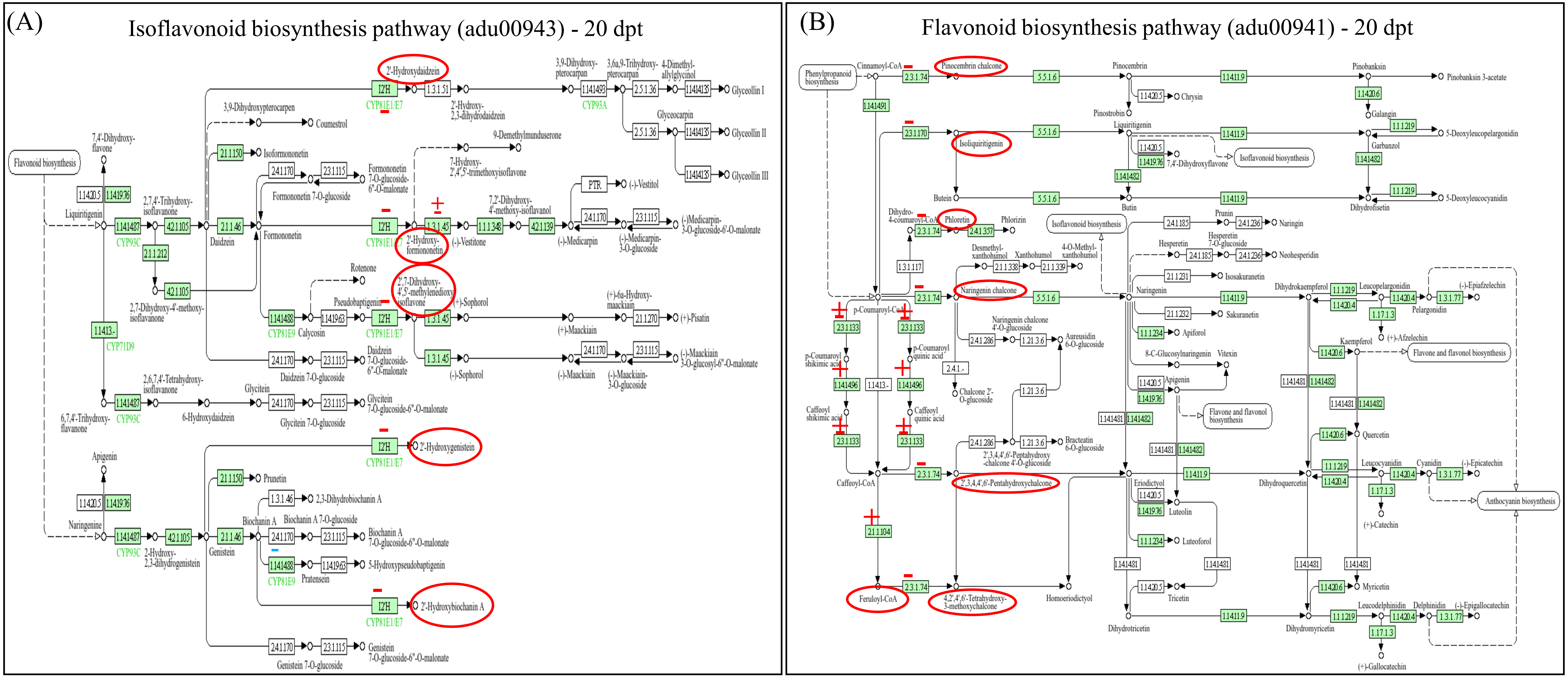

Figure S2. At 20 dpt, Isoflavonoid biosynthesis pathway (adu00943) (A), Flavonoid biosynthesis pathway (adu00941) (B), Circadian rhythm - plant (adu04712) (C), Tropane, piperidine and pyridine alkaloid biosynthesis pathway (adu00960) (D), Biosynthesis of various plant secondary metabolites (adu00999) (E), and Phenylpropanoid biosynthesis pathway (adu00940) (F) were regulated by 0.2, 0.6 and 1.0 treatments. The up- or down- regulated DEGs were represented by "+" or "-", respectively. Red fonts and red ellipses represented the DEGs and circled products were regulated by 0.2, 0.6 and 1.0 treatments.

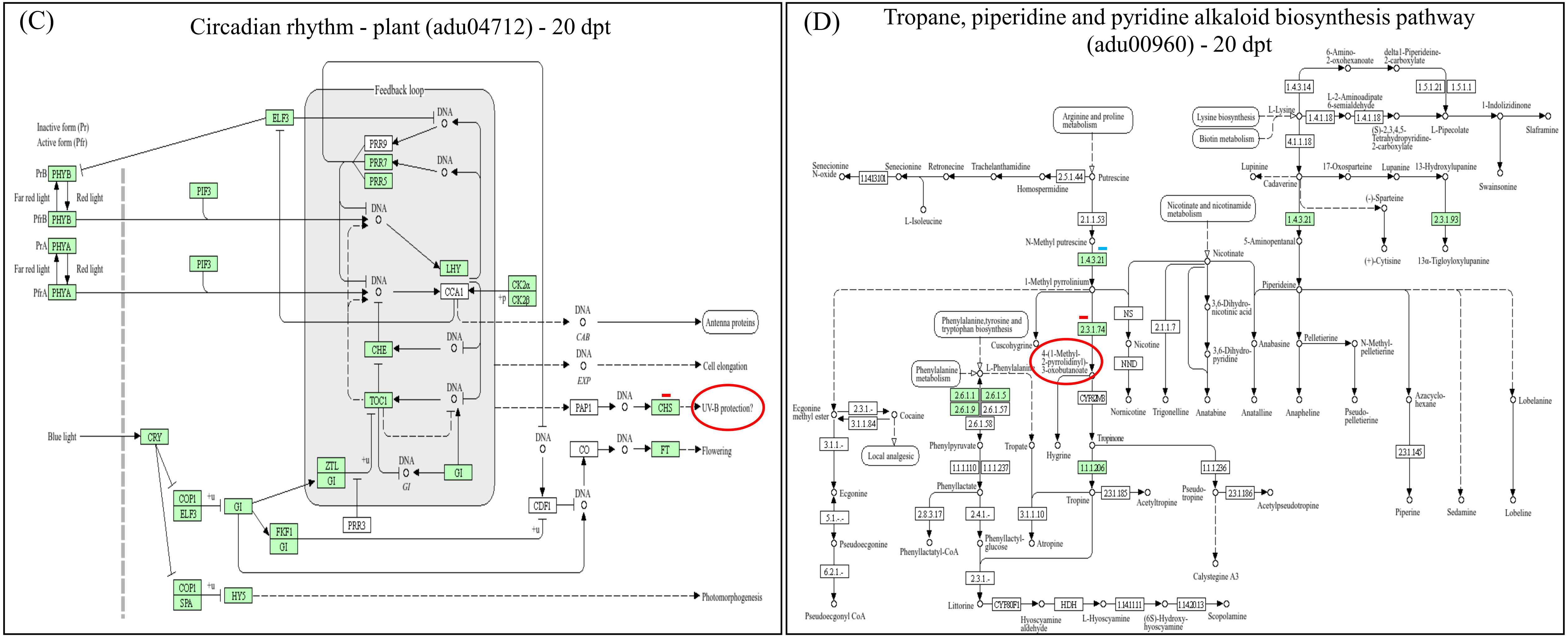
(C)
Circadian rhythm - plant (adu04712) - 20 dpt
Feedback loop
Inactive form (Pr)
Active form (Pfr)
PrB PHYB
Far red light
Red light
PfrB PHYB
PrA PHYA
PfrA PHYA
PIF3
ELF3
PRR9
PRR7
PRR5
DNA
LHY
CCA1
CK2α
CK2β
CHE
TOC1
ZTL
GI
PRR3
FKF1
CDF1
CO
FT
PAP1
CHS
CAB
EXP
Antenna proteins
Cell elongation
UV-B protection?
Flowering
Blue light
CRY
COP1
ELF3
SPA
HY5
Photomorphogenesis
(D)
Tropane, piperidine and pyridine alkaloid biosynthesis pathway (adu00960) - 20 dpt
Arginine and proline metabolism
Putrescine
N-Methyl putrescine
1-Methyl pyrrolinium
4-(1-Methyl-2-pyrrolidinyl)-3-oxobutanoate
Tropinone
Tropine
Pseudotropine
Hygrine
Cuscohygrine
Senecionine N-oxide
Senecionine
Retronecine
Trachelanthamidine
Homospermidine
L-Isoleucine
Phenylalanine,tyrosine and tryptophan biosynthesis
Phenylalanine metabolism
L-Phenylalanine
Phenylpyruvate
Phenyllactate
Phenyllactyl-CoA
Phenyllactyl-glucose
Tropate
Atropine
Littorine
Hyoscyamine aldehyde
L-Hyoscyamine
(6S)-Hydroxy-hyoscyamine
Scopolamine
Acetyltropine
Acetylpseudotropine
Calystegine A3
Ecgonine methyl ester
Cocaine
Local analgesic
Ecgonine
Pseudoecgonine
Pseudoecgonyl CoA
Nicotinate and nicotinamide metabolism
Nicotinate
3,6-Dihydro-nicotinic acid
3,6-Dihydro-pyridine
Nicotine
Nornicotine
Trigonelline
Anatabine
Anabasine
Anatalline
Lysine biosynthesis
Biotin metabolism
L-Lysine
Cadaverine
Lupinine
17-Oxosparteine
Lupanine
13-Hydroxylupanine
13α-Tigloyloxylupanine
(-)-Sparteine
(+)-Cytisine
5-Aminopentanal
Piperideine
Pelletierine
N-Methyl-pelletierine
Anapheline
Pseudo-pelletierine
Azacyclo-hexane
Piperine
Sedamine
Lobelanine
Lobeline
6-Amino-2-oxohexanoate
delta1-Piperideine-2-carboxylate
L-2-Aminoadipate 6-semialdehyde
(S)-2,3,4,5-Tetrahydropyridine-2-carboxylate
L-Pipecolate
1-Indolizidinone
Slaframine
Swainsonine
NS
NND
CYP82M3
CYP80F1
HDH
2.5.1.44
2.1.1.53
1.4.3.21
2.3.1.74
1.1.1.206
2.3.1.185
1.1.1.236
2.3.1.186
2.6.1.1
2.6.1.5
2.6.1.9
2.6.1.57
2.6.1.58
1.1.1.110
1.1.1.237
2.8.3.17
2.4.1.-
3.1.1.10
2.3.1.-
1.14.11.11
1.14.20.13
1.14.13.101
2.1.1.7
2.3.1.93
2.3.1.145
1.4.3.14
1.4.1.18
4.1.1.18
1.5.1.21
1.5.1.1
3.1.1.84
3.1.1.-
5.1.-.-
6.2.1.-

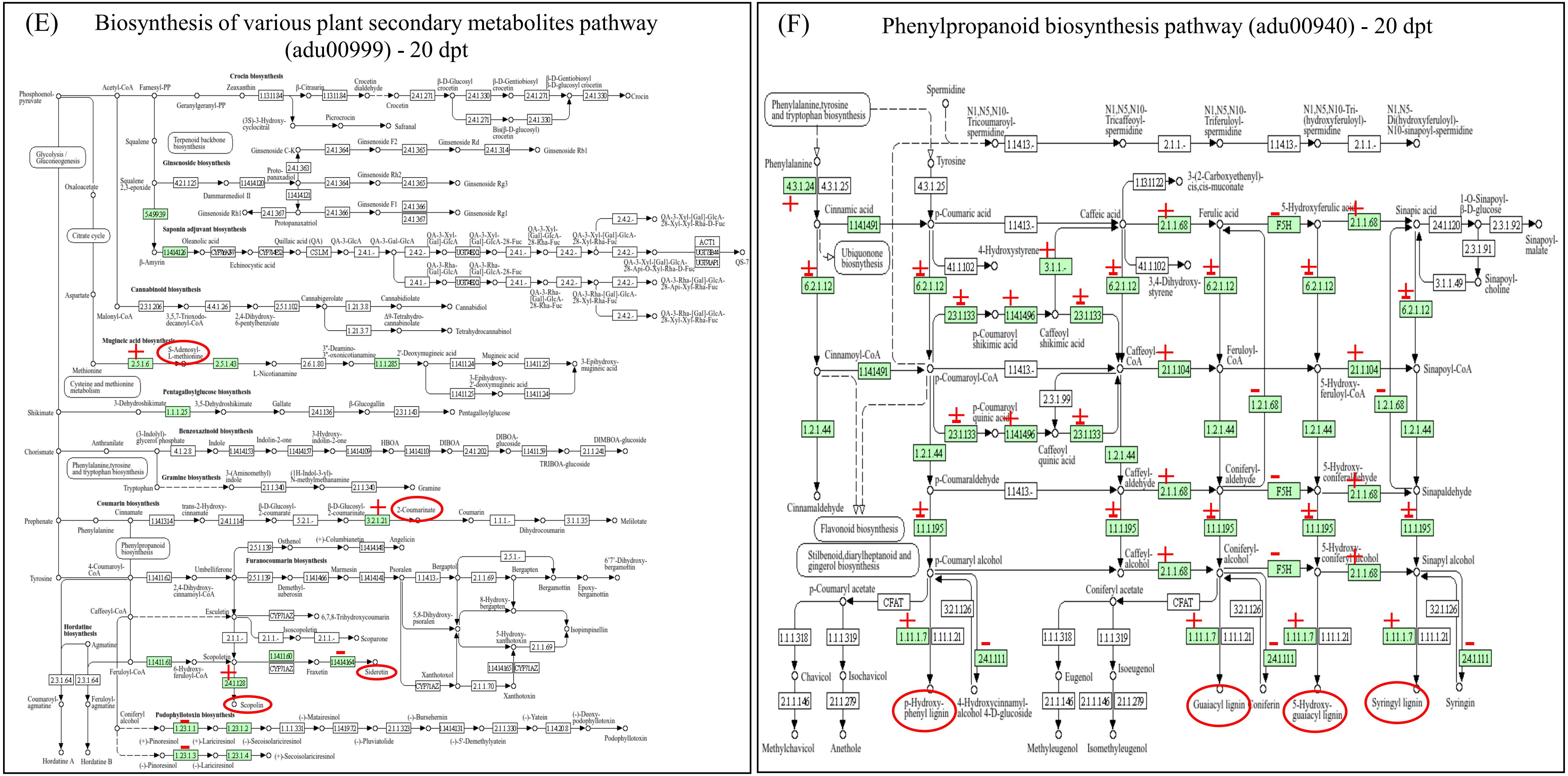
(E) Biosynthesis of various plant secondary metabolites pathway (adu00999) - 20 dpt
Crocin biosynthesis
Phosphoenol-pyruvate
Acetyl-CoA
Farnesyl-PP
Zeaxanthin
β-Citraurin
Crocetin dialdehyde
Crocetin
β-D-Glucosyl crocetin
β-D-Gentiobiosyl crocetin
β-D-Gentiobiosyl β-D-glucosyl crocetin
Crocin
Geranylgeranyl-PP
(3S)-3-Hydroxy-cyclocitral
Picrocrocin
Safranal
Bis(β-D-glucosyl) crocetin
Squalene
Terpenoid backbone biosynthesis
Glycolysis / Gluconeogenesis
Ginsenoside biosynthesis
Ginsenoside C-K
Ginsenoside F2
Ginsenoside Rd
Ginsenoside Rb1
Squalene 2,3-epoxide
Proto-panaxadiol
Dammarenediol II
Ginsenoside Rh2
Ginsenoside Rg3
Oxaloacetate
Ginsenoside Rh1
Ginsenoside F1
Protopanaxatriol
Ginsenoside Rg1
Saponin adjuvant biosynthesis
Citrate cycle
β-Amyrin
Oleanolic acid
Echinocystic acid
Quillaic acid (QA)
QA-3-GlcA
QA-3-Gal-GlcA
Cannabinoid biosynthesis
Aspartate
Malonyl-CoA
3,5,7-Trioxodo-decanoyl-CoA
2,4-Dihydroxy-6-pentylbenzoate
Cannabigerolate
Cannabidiolate
Cannabidiol
Δ9-Tetrahydro-cannabinolate
Tetrahydrocannabinol
Mugineic acid biosynthesis
S-Adenosyl-L-methionine
Methionine
L-Nicotianamine
3''-Deamino-3''-oxonicotianamine
2'-Deoxymugineic acid
Mugineic acid
3-Epihydroxy-mugineic acid
3-Epihydroxy-2'-deoxymugineic acid
Cysteine and methionine metabolism
Pentagalloylglucose biosynthesis
Shikimate
3-Dehydroshikimate
3,5-Dehydroshikimate
Gallate
β-Glucogallin
Pentagalloylglucose
Benzoxazinoid biosynthesis
Chorismate
Anthranilate
(3-Indolyl)-glycerol phosphate
Indole
Indolin-2-one
3-Hydroxy-indolin-2-one
HBOA
DIBOA
DIBOA-glucoside
DIMBOA-glucoside
TRIBOA-glucoside
Phenylalanine,tyrosine and tryptophan biosynthesis
Gramine biosynthesis
Tryptophan
3-(Aminomethyl) indole
(1H-Indol-3-yl)-N-methylmethanamine
Gramine
Coumarin biosynthesis
Prephenate
Phenylalanine
Cinnamate
trans-2-Hydroxy-cinnamate
β-D-Glucosyl-2-coumarate
β-D-Glucosyl-2-coumarinate
2-Coumarinate
Coumarin
Dihydrocoumarin
Melilotate
Phenylpropanoid biosynthesis
Osthenol
(+)-Columbianetin
Angelicin
Furanocoumarin biosynthesis
Tyrosine
4-Coumaroyl-CoA
2,4-Dihydroxy-cinnamoyl-CoA
Umbelliferone
Demethyl-suberosin
Marmesin
Psoralen
Bergaptol
Bergapten
Bergamottin
Epoxy-bergamottin
6',7'-Dihydroxy-bergamottin
Caffeoyl-CoA
Esculetin
6,7,8-Trihydroxycoumarin
Isoscopoletin
Scoparone
5,8-Dihydroxy-psoralen
8-Hydroxy-bergapten
5-Hydroxy-xanthotoxin
Isopimpinellin
Hordatine biosynthesis
Agmatine
Feruloyl-CoA
6-Hydroxy-feruloyl-CoA
Scopoletin
Fraxetin
Sideretin
Scopolin
Xanthotoxol
Xanthotoxin
Coumaroyl-agmatine
Feruloyl-agmatine
Hordatine A
Hordatine B
Coniferyl alcohol
Podophyllotoxin biosynthesis
(+)-Pinoresinol
(+)-Lariciresinol
(-)-Secoisolariciresinol
(-)-Matairesinol
(-)-Pluviatolide
(-)-Bursehernin
(-)-5'-Demethylyatein
(-)-Yatein
(-)-Deoxy-podophyllotoxin
Podophyllotoxin
(-)-Pinoresinol
(-)-Lariciresinol
(+)-Secoisolariciresinol
(F) Phenylpropanoid biosynthesis pathway (adu00940) - 20 dpt
Phenylalanine,tyrosine and tryptophan biosynthesis
Spermidine
N1,N5,N10-Tricoumaroyl-spermidine
N1,N5,N10-Tricaffeoyl-spermidine
N1,N5,N10-Triferuloyl-spermidine
N1,N5,N10-Tri-(hydroxyferuloyl)-spermidine
N1,N5-Di(hydroxyferuloyl)-N10-sinapoyl-spermidine
Phenylalanine
Tyrosine
3-(2-Carboxyethenyl)-cis,cis-muconate
Cinnamic acid
p-Coumaric acid
Caffeic acid
Ferulic acid
5-Hydroxyferulic acid
Sinapic acid
1-O-Sinapoyl-β-D-glucose
Sinapoyl-malate
Ubiquinone biosynthesis
4-Hydroxystyrene
3,4-Dihydroxy-styrene
Sinapoyl-choline
p-Coumaroyl shikimic acid
Caffeoyl shikimic acid
Cinnamoyl-CoA
p-Coumaroyl-CoA
Caffeoyl-CoA
Feruloyl-CoA
5-Hydroxy-feruloyl-CoA
Sinapoyl-CoA
p-Coumaroyl quinic acid
Caffeoyl quinic acid
p-Coumaraldehyde
Caffeyl-aldehyde
Coniferyl-aldehyde
5-Hydroxy-coniferaldehyde
Sinapaldehyde
Cinnamaldehyde
Flavonoid biosynthesis
Stilbenoid,diarylheptanoid and gingerol biosynthesis
p-Coumaryl alcohol
Caffeyl-alcohol
Coniferyl-alcohol
5-Hydroxy-coniferyl alcohol
Sinapyl alcohol
p-Coumaryl acetate
Coniferyl acetate
CFAT
F5H
Chavicol
Isochavicol
Eugenol
Isoeugenol
Methylchavicol
Anethole
Methyleugenol
Isomethyleugenol
p-Hydroxy-phenyl lignin
4-Hydroxycinnamyl-alcohol 4-D-glucoside
Guaiacyl lignin
Coniferin
5-Hydroxy-guaiacyl lignin
Syringyl lignin
Syringin

(A)

(B)

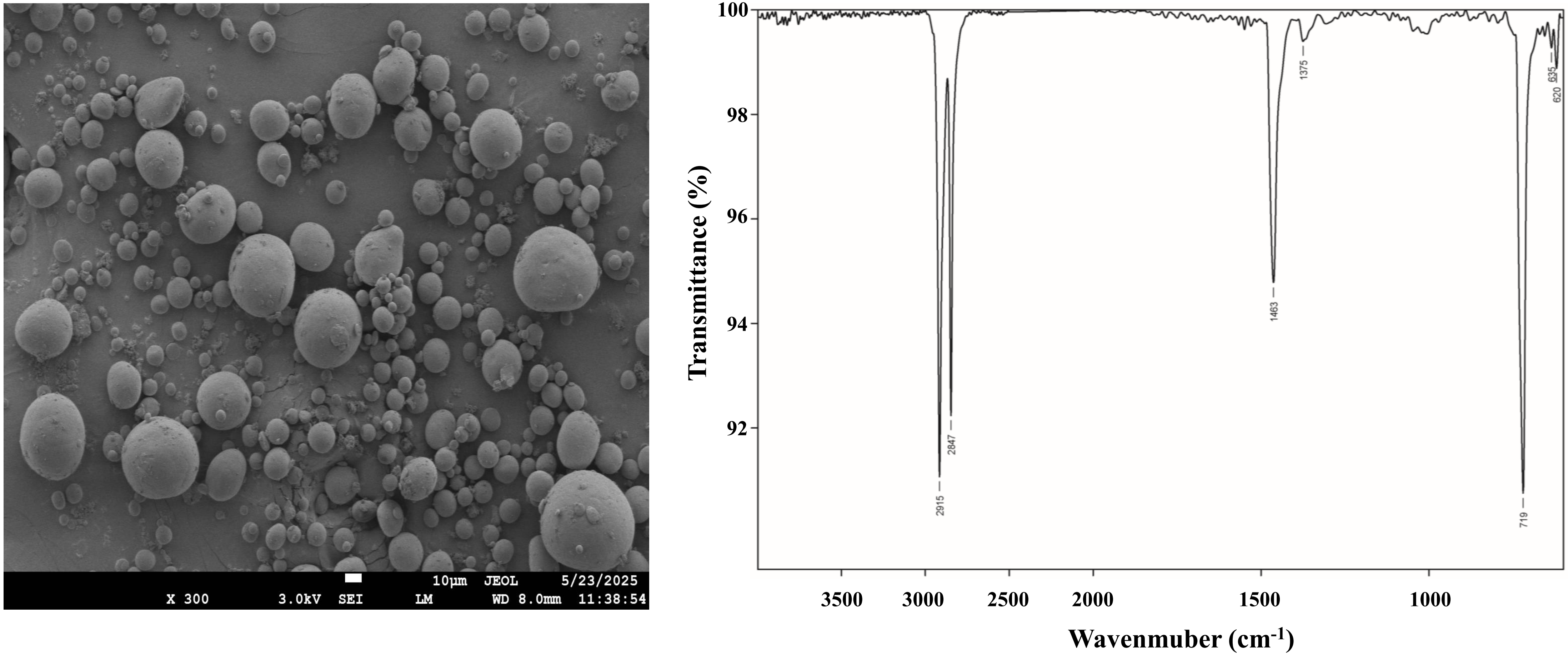

Figure S3. Scanning electron microscopy image (A) and Fourier transform infrared spectrum (B) of PE-MPs.
