# Supplementary material for: Polyethylene Microplastics Inhibit Peanut Nodulation via Metabolic and Transcriptional Pathways
Source: Plants (Basel). 2026 Mar 16;15(6):915. doi: 10.3390/plants15060915 (PMC13030837; doi:10.3390/plants15060915)
Supplement: Supplementary file 1 [file plants-15-00915-s001.zip › Supplementry tables.pdf]

Table S1 Quality of sequencing data.

Sample	Raw reads	Clean reads	Q30 (%)	GC content (%)	Total mapped	Uniquely mapped
Control-1-10 dpt	50637238	49955276	97.68	44.60	46972099 (94.03 %)	39815232 (79.70 %)
Control-2-10 dpt	49293842	48559794	97.65	43.94	46321613 (95.39 %)	39826954 (82.02 %)
Control-3-10 dpt	46046670	45463592	97.64	44.20	43121670 (94.85 %)	37064551 (81.53 %)
0.2-1-10 dpt	48806724	48164446	97.65	44.44	44701490 (92.81 %)	37830103 (78.54 %)
0.2-2-10 dpt	50739184	50038424	97.56	42.84	47095878 (94.12 %)	40079296 (80.10 %)
0.2-3-10 dpt	45203378	44506606	97.59	44.42	42215245 (94.85 %)	36068795 (81.04 %)
0.6-1-10 dpt	45863192	43365646	95.34	43.46	39899100 (92.01 %)	33876947 (78.12 %)
0.6-2-10 dpt	45304068	44519200	97.56	45.36	41161708 (92.46 %)	34552122 (77.61 %)
0.6-3-10 dpt	47183414	46490814	97.57	43.97	44249492 (95.18 %)	38132176 (82.02 %)
1.0-1-10 dpt	43844038	43218154	97.56	44.35	41030896 (94.94 %)	35339209 (81.77 %)
1.0-2-10 dpt	50746788	50032952	97.62	44.75	47053529 (94.05 %)	40148082 (80.24 %)
1.0-3-10 dpt	49122532	48479498	97.68	44.03	46403211 (95.72 %)	40197659 (82.92 %)
Control-1-20 dpt	43421246	42892370	97.58	43.97	41001924 (95.59 %)	35509964 (82.79 %)
Control-2-20 dpt	48117642	47466416	97.72	43.97	45349097 (95.54 %)	38875199 (81.90 %)
Control-3-20 dpt	45487868	44884212	97.53	43.99	42457655 (94.59 %)	36510286 (81.34 %)
0.2-1-20 dpt	43536706	42767424	97.69	44.52	40365098 (94.38 %)	34287367 (80.17 %)
0.2-2-20 dpt	46217262	45624796	97.62	43.96	43417713 (95.16 %)	37302535 (81.76 %)
0.2-3-20 dpt	46217262	45720888	97.55	44.08	43719921 (95.62 %)	37503703 (82.03 %)
0.6-1-20 dpt	46695462	46039842	97.61	44.00	44377858 (96.39 %)	38381119 (83.37 %)
0.6-2-20 dpt	50755200	49887930	97.65	44.23	45250340 (90.70 %)	38808800 (77.79 %)
0.6-3-20 dpt	44324164	43652666	97.62	44.11	42147855 (96.55 %)	36429739 (83.45 %)
1.0-1-20 dpt	41984126	41419856	97.67	44.32	39328062 (94.95 %)	33682792 (81.32 %)
1.0-2-20 dpt	41005270	40447254	97.54	44.03	38220704 (94.50 %)	32702179 (80.85 %)
1.0-3-20 dpt	45920696	45288514	97.62	43.90	43007939 (94.96 %)	36971154 (81.63 %)

Table S2 Statistical information of GO annotated DEGs.

Comparison Group	Category	GO ID	Count	Description	Gene Ratio	BgRatio	p value	p adj
0.2 vs. Control-10 dpt	BP	GO:0006022	11	Aminoglycan metabolic process	11/691	21/13829	9.99E-10	3.54E-08
	BP	GO:0006026	11	Aminoglycan catabolic process	11/691	21/13829	9.99E-10	3.54E-08
	BP	GO:0006030	11	Chitin metabolic process	11/691	21/13829	9.99E-10	3.54E-08
	BP	GO:0006032	11	Chitin catabolic process	11/691	21/13829	9.99E-10	3.54E-08
	CC	GO:0034357	22	Photosynthetic membrane	22/214	80/4916	1.13E-12	5.50E-11
	CC	GO:0009579	22	Thylakoid	22/214	83/4916	2.58E-12	5.50E-11
	CC	GO:0044436	22	Thylakoid part	22/214	83/4916	2.58E-12	5.50E-11
	CC	GO:0009521	20	Photosystem	20/214	76/4916	3.12E-11	4.99E-10
	MF	GO:0016705	58	Oxidoreductase activity, acting on paired donors, with incorporation or reduction of molecular oxygen	58/1062	490/22798	7.26E-11	2.00E-08
	MF	GO:0004568	11	Chitinase activity	11/1062	21/22798	4.89E-10	6.72E-08
	MF	GO:0005506	54	Iron ion binding	54/1062	496/22798	6.67E-09	6.11E-07
	MF	GO:0016701	17	Oxidoreductase activity, acting on single donors with incorporation of molecular oxygen	17/1062	97/22798	2.25E-06	0.0001436
0.6 vs. Control-10 dpt	BP	GO:0016567	19	Protein ubiquitination	19/559	115/13999	1.33E-07	2.75E-05
	BP	GO:0032446	19	Protein modification by small protein conjugation	19/559	117/13999	1.77E-07	2.75E-05
	BP	GO:0070647	20	Protein modification by small protein conjugation or removal	20/559	180/13999	3.42E-05	0.0035486
	BP	GO:0006952	14	Defense response	14/559	138/13999	0.00124	0.0437384
	CC	GO:0005576	11	Extracellular region	11/114	72/4974	5.27E-07	1.95E-05
	CC	GO:0031012	5	Extracellular matrix	5/114	14/4974	9.83E-06	0.0001213
	CC	GO:0044421	5	Extracellular region part	5/114	14/4974	9.83E-06	0.0001213
	CC	GO:0071944	15	Cell periphery	15/114	182/4974	1.38E-05	0.0001274
	MF	GO:0004842	24	Ubiquitin-protein transferase activity	24/871	160/23033	8.44E-09	9.16E-07
	MF	GO:0019787	24	Ubiquitin-like protein transferase activity	24/871	160/23033	8.44E-09	9.16E-07
	MF	GO:0005509	29	Calcium ion binding	29/871	311/23033	8.10E-06	0.000586
	MF	GO:0043565	26	Sequence-specific DNA binding	26/871	336/23033	0.00048	0.0261249
1.0 vs. Control-10 dpt	BP	GO:0016567	37	Protein ubiquitination	37/1396	117/14083	6.92E-11	2.97E-08
	BP	GO:0032446	37	Protein modification by small protein conjugation	37/1396	119/14083	1.20E-10	2.97E-08
	BP	GO:0009733	19	Response to auxin	19/1396	55/14083	6.42E-07	0.0001057
	BP	GO:0006887	25	Exocytosis	25/1396	104/14083	2.13E-05	0.0017566
	CC	GO:0000145	25	Exocyst	25/307	79/4986	2.52E-12	4.46E-11
	CC	GO:0005938	25	Cell cortex	25/307	79/4986	2.52E-12	4.46E-11
	CC	GO:0044448	25	Cell cortex part	25/307	79/4986	2.52E-12	4.46E-11
	CC	GO:0099568	25	Cytoplasmic region	25/307	79/4986	2.52E-12	4.46E-11
	MF	GO:0043565	73	Sequence-specific DNA binding	73/2133	337/23160	3.26E-12	1.02E-09
	MF	GO:0004842	42	Ubiquitin-protein transferase activity	42/2133	162/23160	4.38E-10	4.55E-08
	MF	GO:0019787	42	Ubiquitin-like protein transferase activity	42/2133	162/23160	4.38E-10	4.55E-08
	MF	GO:0005509	64	Calcium ion binding	64/2133	315/23160	1.13E-09	8.85E-08
0.2 vs. Control-20 dpt	BP	GO:0042737	14	Drug catabolic process	14/2010	24/13897	7.87E-07	0.0002355
	BP	GO:0051704	53	Multi-organism process	53/2010	192/13897	1.45E-06	0.0002355
	BP	GO:0008037	50	Cell recognition	50/2010	181/13897	2.78E-06	0.0002355
	BP	GO:0009856	50	Pollination	50/2010	181/13897	2.78E-06	0.0002355
	CC	GO:0005840	88	Ribosome	88/603	449/4936	1.69E-06	0.000204
	MF	GO:0016705	123	Oxidoreductase activity, acting on paired donors, with incorporation or reduction of molecular oxygen	123/2949	497/22896	2.95E-13	1.09E-10
	MF	GO:0043565	78	Sequence-specific DNA binding	78/2949	333/22896	8.11E-08	1.49E-05
	MF	GO:0016684	50	Oxidoreductase activity, acting on	50/2949	194/22896	8.82E-07	8.62E-05
	MF	GO:0004601	49	Peroxidase activity	49/2949	189/22896	9.37E-07	8.62E-05
0.6 vs. Control-20 dpt	BP	GO:0015979	41	Photosynthesis	41/2300	90/13975	8.78E-11	5.01E-08
	BP	GO:0071554	49	Cell wall organization or biogenesis	49/2300	148/13975	4.63E-07	0.0001322
	BP	GO:0045229	39	External encapsulating structure	39/2300	118/13975	6.98E-06	0.0009113
	BP	GO:0071555	39	Cell wall organization	39/2300	118/13975	6.98E-06	0.0009113
	CC	GO:0009579	45	Thylakoid	45/735	82/4963	1.77E-17	1.21E-15
	CC	GO:0044436	45	Thylakoid part	45/735	82/4963	1.77E-17	1.21E-15

	CC	GO:0034357	43	Photosynthetic membrane	43/735	79/4963	1.42E-16	6.48E-15
	CC	GO:0009521	41	Photosystem	41/735	75/4963	6.03E-16	2.06E-14
	MF	GO:0004252	50	Serine-type endopeptidase activity	50/3461	150/23049	1.55E-08	5.90E-06
	MF	GO:0008171	34	O-methyltransferase activity	34/3461	92/23049	1.89E-07	3.58E-05
	MF	GO:0043565	85	Sequence-specific DNA binding	85/3461	333/23049	3.48E-07	4.41E-05
	MF	GO:0004857	61	Enzyme inhibitor activity	61/3461	218/23049	5.87E-07	5.58E-05
1.0 vs. Control-20 dpt	BP	GO:0008037	57	Cell recognition	57/1730	180/13966	6.69E-12	7.88E-10
	BP	GO:0009856	57	Pollination	57/1730	180/13966	6.69E-12	7.88E-10
	BP	GO:0009875	57	Pollen-pistil interaction	57/1730	180/13966	6.69E-12	7.88E-10
	BP	GO:0044706	57	Multi-multicellular organism process	57/1730	180/13966	6.69E-12	7.88E-10
	CC	GO:0034357	23	Photosynthetic membrane	23/492	80/4972	1.47E-06	8.76E-05
	CC	GO:0009521	22	Photosystem	22/492	76/4972	2.20E-06	8.76E-05
	CC	GO:0009579	23	Thylakoid	23/492	82/4972	2.37E-06	8.76E-05
	CC	GO:0044436	23	Thylakoid part	23/492	82/4972	2.37E-06	8.76E-05
	MF	GO:0016705	98	Oxidoreductase activity, acting on paired donors, with incorporation or reduction of molecular oxygen	98/2544	500/23033	1.03E-08	3.76E-06
	MF	GO:0004674	25	Protein serine/threonine kinase activity	25/2544	77/23033	3.95E-07	7.19E-05
	MF	GO:0043565	67	Sequence-specific DNA binding	67/2544	333/23033	8.11E-07	9.84E-05
	MF	GO:0016759	22	Cellulose synthase activity	22/2544	69/23033	2.73E-06	0.0001985

Table S3 Statistical information of KEGG annotated DEGs.

Comparison Group	KEGG ID	Count	Up	Down	Description	Gene Ratio	Bg Ratio	p value	p adj
0.2 vs. Control-10 dpt	adu00196	19	2	17	Photosynthesis - antenna proteins	19/472	34/11188	5.48E-18	6.24E-16
	adu00195	25	0	25	Photosynthesis	25/472	93/11188	4.77E-14	2.72E-12
	adu00943	11	8	3	Isoflavonoid biosynthesis	11/472	51/11188	6.91E-06	0.0002625
	adu00710	19	5	14	Carbon fixation in photosynthetic organisms	19/472	154/11188	2.53E-05	0.0007203
	adu00941	16	14	2	Flavonoid biosynthesis	16/472	132/11188	0.000135	0.0030709
	adu04712	16	11	5	Circadian rhythm - plant	16/472	141/11188	0.000291	0.0055265
	adu00904	8	5	3	Diterpenoid biosynthesis	8/472	57/11188	0.00058	0.0094492
	adu00040	17	5	12	Pentose and glucuronate interconversions	17/472	185/11188	0.000821	0.0116998
	adu00910	9	2	7	Nitrogen metabolism	9/472	63/11188	0.001221	0.015462
	adu00960	12	12	0	Tropane, piperidine and pyridine alkaloid biosynthesis	12/472	110/11188	0.002277	0.025962
	adu00592	11	7	4	alpha-Linolenic acid metabolism	11/472	104/11188	0.004344	0.0450218
0.6 vs. Control-10 dpt	adu00941	43	43	0	Flavonoid biosynthesis	43/372	135/11318	3.58E-31	3.97E-29
	adu00960	40	40	0	Tropane, piperidine and pyridine alkaloid biosynthesis	40/372	116/11318	1.37E-30	7.58E-29
	adu04712	37	37	0	Circadian rhythm - plant	37/372	144/11318	3.09E-23	1.15E-21
	adu00943	16	14	2	Isoflavonoid biosynthesis	16/372	55/11318	1.24E-11	3.45E-10
	adu04016	28	20	8	MAPK signaling pathway - plant	28/372	292/11318	3.59E-07	7.97E-06
	adu04626	32	29	3	Plant-pathogen interaction	32/372	398/11318	2.65E-06	4.90E-05
	adu00999	14	13	1	Biosynthesis of various plant secondary metabolites	14/372	113/11318	1.92E-05	0.0003046
	adu00940	19	10	9	Phenylpropanoid biosynthesis	19/372	288/11318	0.003102	0.0430343
1.0 vs. Control-10 dpt	adu00941	48	46	2	Flavonoid biosynthesis	48/798	138/11328	1.12E-21	1.398E-19
	adu00960	43	38	5	Tropane, piperidine and pyridine alkaloid biosynthesis	43/798	115/11328	5.91E-21	3.695E-19
	adu04712	42	40	2	Circadian rhythm - plant	42/798	144/11328	5.96E-16	2.483E-14
	adu04626	72	67	5	Plant-pathogen interaction	72/798	407/11328	1.69E-13	5.279E-12
	adu04016	57	39	18	MAPK signaling pathway - plant	57/798	303/11328	4.98E-12	1.244E-10
	adu00943	15	14	1	Isoflavonoid biosynthesis	15/798	54/11328	2.95E-06	6.149E-05
	adu00750	9	7	2	Vitamin B6 metabolism	9/798	29/11328	0.000113	0.0020212
	adu01250	29	22	7	Biosynthesis of nucleotide sugars	29/798	217/11328	0.000626	0.0097748
	adu00592	17	13	4	alpha-Linolenic acid metabolism	17/798	105/11328	0.001007	0.0139859
	adu00940	35	22	13	Phenylpropanoid biosynthesis	35/798	291/11328	0.00128	0.016003
	adu00460	15	8	7	Cyanoamino acid metabolism	15/798	92/11328	0.001817	0.0206485
	adu00400	17	10	7	Phenylalanine, tyrosine and tryptophan biosynthesis	17/798	114/11328	0.002522	0.0262678
	adu00908	10	5	5	Zeatin biosynthesis	10/798	55/11328	0.004576	0.0439987
0.2 vs. Control-20 dpt	adu00940	90	62	28	Phenylpropanoid biosynthesis	90/1345	295/11245	5.72E-18	7.38E-16
	adu04712	47	7	40	Circadian rhythm - plant	47/1345	143/11245	3.11E-11	2.00E-09
	adu00941	44	14	30	Flavonoid biosynthesis	44/1345	135/11245	1.81E-10	7.77E-09
	adu00480	47	16	31	Glutathione metabolism	47/1345	222/11245	5.86E-05	0.0015151
	adu02010	40	11	29	ABC transporters	40/1345	179/11245	5.87E-05	0.0015151
	adu00999	27	16	11	Biosynthesis of various plant secondary metabolites	27/1345	111/11245	0.000212	0.0045558
	adu00943	16	7	9	Isoflavonoid biosynthesis	16/1345	53/11245	0.000316	0.0058175
	adu00966	8	1	7	Glucosinolate biosynthesis	8/1345	17/11245	0.000368	0.005932
	adu00906	18	16	2	Carotenoid biosynthesis	18/1345	65/11245	0.000446	0.0063938
	adu00270	46	33	13	Cysteine and methionine metabolism	46/1345	238/11245	0.000621	0.0080159
	adu00260	30	22	8	Glycine, serine and threonine metabolism	30/1345	139/11245	0.000869	0.0101933
	adu00130	29	16	13	Ubiquinone and other terpenoid-quinone biosynthesis	29/1345	134/11245	0.001004	0.010796
	adu00960	26	2	24	Tropane, piperidine and pyridine alkaloid biosynthesis	26/1345	117/11245	0.001194	0.0118485
	adu00920	19	11	8	Sulfur metabolism	19/1345	78/11245	0.00172	0.0158494
	adu00904	14	13	1	Diterpenoid biosynthesis	14/1345	53/11245	0.003031	0.0260687
	adu00591	13	1	12	Linoleic acid metabolism	13/1345	50/11245	0.004879	0.039333
	adu00520	52	47	5	Amino sugar and nucleotide sugar metabolism	52/1345	307/11245	0.005726	0.0434491
0.6 vs. Control-20 dpt	adu00941	63	14	49	Flavonoid biosynthesis	63/1441	133/11310	9.72E-23	1.25E-20
	adu00195	50	49	1	Photosynthesis	50/1441	93/11310	2.09E-21	1.35E-19
	adu00999	45	30	15	Biosynthesis of various plant secondary metabolites	45/1441	112/11310	2.31E-13	9.95E-12
	adu00196	22	22	0	Photosynthesis - antenna proteins	22/1441	34/11310	2.12E-12	6.82E-11
	adu00960	42	1	41	Tropane, piperidine and pyridine alkaloid biosynthesis	42/1441	113/11310	2.98E-11	7.70E-10
	adu04712	46	6	40	Circadian rhythm - plant	46/1441	143/11310	9.55E-10	2.05E-08
	adu00940	73	54	19	Phenylpropanoid biosynthesis	73/1441	293/11310	6.96E-09	1.28E-07
	adu00040	47	39	8	Pentose and glucuronate interconversions	47/1441	181/11310	9.63E-07	1.55E-05

	adu00945	16	12	4	Stilbenoid, diarylheptanoid and gingerol biosynthesis	16/1441	38/11310	6.26E-06	8.97E-05
	adu00500	63	52	11	Starch and sucrose metabolism	63/1441	321/11310	0.000263	0.0033877
	adu00460	23	21	2	Cyanoamino acid metabolism	23/1441	92/11310	0.000975	0.011431
	adu00943	16	2	14	Isoflavonoid biosynthesis	16/1441	56/11310	0.00125	0.0134409
	adu00908	15	6	9	Zeatin biosynthesis	15/1441	55/11310	0.002903	0.0288046
	adu00966	7	1	6	Glucosinolate biosynthesis	7/1441	17/11310	0.003263	0.0300685
	adu04814	44	35	9	Motor proteins	44/1441	235/11310	0.005229	0.0449669
1.0 vs. Control-20 dpt	adu00480	52	17	35	Glutathione metabolism	52/1119	221/11287	1.97E-09	2.55E-07
	adu00941	36	10	26	Flavonoid biosynthesis	36/1119	138/11287	3.75E-08	2.42E-06
	adu00195	27	27	0	Photosynthesis	27/1119	93/11287	1.81E-07	7.79E-06
	adu00940	56	32	24	Phenylpropanoid biosynthesis	56/1119	293/11287	9.84E-07	3.17E-05
	adu00196	13	13	0	Photosynthesis - antenna proteins	13/1119	34/11287	1.06E-05	0.0002731
	adu00904	17	16	1	Diterpenoid biosynthesis	17/1119	56/11287	1.77E-05	0.0003802
	adu00250	27	20	7	Alanine, aspartate and glutamate metabolism	27/1119	123/11287	5.62E-05	0.0010355
	adu02010	35	10	25	ABC transporters	35/1119	181/11287	8.13E-05	0.0013116
	adu00906	17	15	2	Carotenoid biosynthesis	17/1119	65/11287	0.000143	0.0020445
	adu00943	15	6	9	Isoflavonoid biosynthesis	15/1119	56/11287	0.000261	0.0033655
	adu00999	23	17	6	Biosynthesis of various plant secondary metabolites	23/1119	110/11287	0.000412	0.0048346
	adu00591	13	2	11	Linoleic acid metabolism	13/1119	49/11287	0.000731	0.0078569
	adu00010	46	34	12	Glycolysis / Gluconeogenesis	46/1119	299/11287	0.001663	0.0165024
	adu00960	22	4	18	Tropane, piperidine and pyridine alkaloid biosynthesis	22/1119	117/11287	0.00235	0.0216564
	adu00920	16	12	4	Sulfur metabolism	16/1119	77/11287	0.003182	0.0273609
	adu00945	10	8	2	Stilbenoid, diarylheptanoid and gingerol biosynthesis	10/1119	39/11287	0.003848	0.0310238
	adu04712	25	7	18	Circadian rhythm - plant	25/1119	148/11287	0.005489	0.0416487
	adu00710	25	23	2	Carbon fixation in photosynthetic organisms	25/1119	150/11287	0.006535	0.0468322

Table S4 Gene expression levels of KEGG annotated DEGs of 0.2, 0.6, and 1.0 treatments compared with the Control at 10 dpt.

Pathway	KEGG ID	Gene ID	0.2	0.6	1.0	Product
Isoflavonoid biosynthesis	adu00943	112740691	+	ns	+	
		112741794	+	+	ns	
		112791520	+	+	+	2-hydroxyisoflavanone synthase
		112720154	-	ns	ns	
		112791519	+	+	+	isoflavone 4'-O-methyltransferase
		112696146	+	+	+	pterocarpan synthase
		112792439	+	+	+	isoflavone/4'-methoxyisoflavone 2'-hydroxylase
		112716970	+	ns	ns	
		112790914	-	ns	ns	
		112800734	+	+	+	2'-hydroxyisoflavone reductase
		112709830	-	ns	ns	
		112799808	ns	+	+	
		112797857	ns	+	ns	
		112734659	ns	+	+	
		112748459	ns	+	ns	
		112758883	ns	+	+	
		112733553	ns	+	ns	
		112798104	ns	-	-	
		112749381	ns	+	ns	
		112734657	ns	-	ns	
		112733554	ns	+	+	
		112707909	ns	ns	+	
		112800541	ns	ns	+	
		112796477	ns	ns	+	
		112741792	ns	ns	+	
Flavonoid biosynthesis	adu00041	112777310	+	ns	+	
		112712108	+	+	+	trans-cinnamate 4-monooxygenase
		112789292	+	+	+	chalcone synthase
		112740947	+	+	+	chalcone synthase
		112795670	+	+	+	chalcone synthase
		112741594	+	+	+	chalcone synthase
		112796277	+	+	+	chalcone synthase
		112785158	-	ns	ns	
		112796274	+	+	+	chalcone synthase
		112701703	+	ns	+	
		112764075	+	ns	ns	
		112741585	+	+	+	chalcone synthase
		112796266	+	+	+	chalcone synthase
		112796256	+	+	+	chalcone synthase
		112710267	-	ns	ns	
		112741570	+	+	+	chalcone synthase
		112766362	ns	+	+	
		112741581	ns	+	+	
		112701702	ns	+	+	
		112796265	ns	+	+	
		112796255	ns	+	+	
		112741586	ns	+	+	
		112741579	ns	+	+	
		112741580	ns	+	+	
		112796275	ns	+	+	
		112741559	ns	+	+	
		112741590	ns	+	+	
		112755723	ns	+	+	
		112720208	ns	+	+	
		112790504	ns	+	+	
		112741543	ns	+	+	
		112741555	ns	+	+	

		112741561	ns	+	+	
		112796261	ns	+	+	
		112796259	ns	+	+	
		112796258	ns	+	+	
		112796273	ns	+	+	
		112794644	ns	+	+	
		112796253	ns	+	+	
		112802037	ns	+	+	
		112741558	ns	+	ns	
		112796278	ns	+	+	
		112755722	ns	+	+	
		112796251	ns	+	+	
		112696965	ns	+	ns	
		112741591	ns	+	+	
		112765123	ns	+	+	
		112741589	ns	+	+	
		112799008	ns	ns	-	
		112764643	ns	ns	-	
		112741587	ns	ns	+	
		112796257	ns	ns	+	
		112719684	ns	ns	+	
Circadian rhythm - plant	adu04712	112707413	-	ns	ns	
		112747847	+	ns	ns	
		112770735	-	ns	ns	
		112789292	+	+	+	chalcone synthase
		112740947	+	+	+	chalcone synthase
		112795670	+	+	+	chalcone synthase
		112741594	+	+	+	chalcone synthase
		112796277	+	+	+	chalcone synthase
		112757971	-	ns	ns	
		112695653	-	ns	-	
		112796274	+	+	+	chalcone synthase
		112695628	-	ns	ns	
		112741585	+	+	+	chalcone synthase
		112796266	+	+	+	chalcone synthase
		112796256	+	+	+	chalcone synthase
		112741570	+	+	+	chalcone synthase
		112796265	ns	+	+	
		112741581	ns	+	+	
		112796255	ns	+	+	
		112741586	ns	+	+	
		112741579	ns	+	+	
		112741580	ns	+	+	
		112796275	ns	+	+	
		112741559	ns	+	+	
		112741590	ns	+	+	
		112755723	ns	+	+	
		112720208	ns	+	+	
		112790504	ns	+	+	
		112741543	ns	+	+	
		112741555	ns	+	+	
		112741561	ns	+	+	
		112796261	ns	+	+	
		112796259	ns	+	+	
		112796258	ns	+	+	
		112796273	ns	+	+	
		112794644	ns	+	+	
		112796253	ns	+	+	
		112741558	ns	+	ns	
		112796278	ns	+	+	
		112755722	ns	+	+	

		112796251	ns	+	+	
		112741591	ns	+	+	
		112741589	ns	+	+	
		112747841	ns	ns	-	
		112775500	ns	ns	+	
		112712245	ns	ns	+	
		112741587	ns	ns	+	
		112796257	ns	ns	-	
Tropane, piperidine and pyridine alkaloid biosynthesis	adu00960	112789292	+	+	+	chalcone synthase
		112740947	+	+	+	chalcone synthase
		112795670	+	+	+	chalcone synthase
		112741594	+	+	+	chalcone synthase
		112796277	+	+	+	chalcone synthase
		112778213	+	ns	ns	
		112796274	+	+	+	chalcone synthase
		112741585	+	+	+	chalcone synthase
		112796266	+	+	+	chalcone synthase
		112796256	+	+	+	chalcone synthase
		112789831	+	ns	ns	
		112741570	+	+	+	chalcone synthase
		112741581	ns	+	+	
		112796265	ns	+	+	
		112796255	ns	+	+	
		112741586	ns	+	+	
		112741579	ns	+	+	
		112741580	ns	+	+	
		112796275	ns	+	+	
		112741559	ns	+	+	
		112741590	ns	+	+	
		112755723	ns	+	+	
		112736707	ns	+	ns	
		112720208	ns	+	+	
		112790504	ns	+	+	
		112741543	ns	+	+	
		112741555	ns	+	+	
		112741561	ns	+	+	
		112796261	ns	+	+	
		112796259	ns	+	+	
		112796258	ns	+	+	
		112796273	ns	+	+	
		112794644	ns	+	+	
		112796253	ns	+	+	
		112741558	ns	+	ns	
		112796278	ns	+	+	
		112711799	ns	+	ns	
		112755722	ns	+	+	
		112796251	ns	+	+	
		112796915	ns	+	ns	
		112741591	ns	+	+	
		112741589	ns	+	+	
		112737991	ns	ns	-	
		112790135	ns	ns	-	
		112790449	ns	ns	-	
		112736288	ns	ns	-	
		112801699	ns	ns	-	
		112741587	ns	ns	+	
		112796257	ns	ns	+	

"+": Positive, compared to the Control, the gene expression level was increased; "-": Negative, compared to the control, the gene expression level was decreased; "ns": not significant, compared with the Control, the gene expression was not significant.

Table S5 Gene expression levels of KEGG annotated DEGs of 0.2, 0.6, and 1.0 treatments compared with the Control at 20 dpt.

Pathway	KEGG ID	Gene ID	0.2	0.6	1.0	Product
Isoflavonoid biosynthesis	adu00943	112711824	+	ns	+	
		112776333	+	ns	+	
		112734657	+	ns	ns	
		112749381	-	-	-	2'-hydroxyisoflavone reductase
		112748468	-	ns	-	
		112733553	+	-	+	
		112796477	-	-	-	isoflavone/4'-methoxyisoflavone [illegible]
		112800537	-	ns	ns	
		112800734	-	-	-	2'-hydroxyisoflavone reductase
		112720547	-	ns	ns	
		112748463	+	+	+	isoflavone/4'-methoxyisoflavone [illegible]
		112744553	-	-	ns	
		112720154	-	ns	ns	
		112741792	-	-	-	isoflavone/4'-methoxyisoflavone [illegible]
		112801706	+	ns	+	
		112744581	+	ns	+	
		112740936	ns	+	ns	
		112741794	ns	-	-	
		112716970	ns	-	ns	
		112696146	ns	-	ns	
		112740687	ns	-	ns	
		112794481	ns	-	-	
		112800534	ns	-	-	
		112791519	ns	-	ns	
		112748459	ns	-	ns	
Flavonoid biosynthesis	adu00941	112796254	-	-	-	chalcone synthase
		112782895	+	+	+	caffeoyl-CoA O-methyltransferase
		112715608	+	+	+	caffeoyl-CoA O-methyltransferase
		112784679	+	+	+	caffeoyl-CoA O-methyltransferase
		112696779	+	+	+	5-O-(4-coumaroyl)-D-quinate
		112790504	-	-	-	chalcone synthase
		112754637	+	+	+	5-O-(4-coumaroyl)-D-quinate
		112720208	-	-	-	chalcone synthase
		112791159	-	ns	ns	
		112709716	+	+	+	shikimate O-hydroxycinnamoyltransferase
		112780096	+	+	+	shikimate O-hydroxycinnamoyltransferase
		112741555	-	-	-	chalcone synthase
		112741561	-	-	-	chalcone synthase
		112796273	-	ns	-	
		112801348	+	ns	+	
		112796275	-	-	ns	
		112712326	+	ns	+	
		112740947	-	-	-	chalcone synthase
		112741589	-	-	-	chalcone synthase
		112796274	-	-	ns	
		112795670	-	-	-	chalcone synthase
		112741594	-	-	ns	
		112775408	+	ns	ns	
		112738206	-	-	-	chalcone reductase
		112796379	-	ns	-	
		112741559	-	-	-	chalcone reductase
		112712329	+	ns	ns	
		112777442	-	ns	-	
		112712327	+	ns	ns	
		112796255	-	-	ns	
		112719684	-	ns	ns	
		112741585	-	-	ns	
		112796265	-	ns	-	
		112701712	-	ns	ns	

		112796256	-	-	-	chalcone reductase
		112764966	-	ns	ns	
		112754846	-	-	-	shikimate O-hydroxycinnamoyltransferase
		112796266	-	-	ns	
		112796258	-	-	-	chalcone synthase
		112697083	+	+	ns	
		112796278	-	-	-	chalcone synthase
		112719871	+	ns	ns	
		112741574	-	-	ns	
		112741590	-	-	-	chalcone synthase
		112799024	ns	+	ns	
		112799008	ns	+	+	caffeoyl-CoA O-methyltransferase
		112720495	ns	+	ns	
		112728650	ns	+	ns	
		112715071	ns	+	ns	
		112720490	ns	+	ns	
		112796253	ns	-	ns	
		112740948	ns	-	ns	
		112755723	ns	-	ns	
		112701704	ns	-	-	chalcone reductase
		112741553	ns	-	-	chalcone reductase
		112796277	ns	-	ns	
		112796259	ns	-	ns	
		112697592	ns	-	-	chalcone reductase
		112741581	ns	-	ns	
		112741570	ns	-	ns	
		112741580	ns	-	ns	
		112755722	ns	-	ns	
		112741542	ns	-	ns	
		112701703	ns	-	ns	
		112764077	ns	-	ns	
		112741543	ns	-	ns	
		112764075	ns	-	ns	
		112790341	ns	-	-	chalcone reductase
		112741586	ns	-	ns	
		112696965	ns	-	ns	
		112741591	ns	-	-	chalcone reductase
		112745809	ns	-	ns	
		112796251	ns	-	ns	
		112727631	ns	-	ns	
		112796261	ns	-	ns	
		112796257	ns	-	ns	
		112741566	ns	-	ns	
		112764077	ns	ns	-	
		112696962	ns	ns	-	
Circadian rhythm - plant	adu04712	112796254	-	-	-	chalcone synthase
		112764504	-	ns	-	
		112790504	-	-	-	chalcone synthase
		112720208	-	-	-	chalcone synthase
		112706385	-	ns	ns	
		112758575	+	ns	+	
		112741555	-	-	-	chalcone synthase
		112741561	-	-	-	chalcone synthase
		112796273	-	ns	-	
		112801348	+	ns	+	
		112695249	+	ns	+	
		112796275	-	-	ns	
		112782919	-	ns	ns	
		112734552	+	ns	+	
		112740947	-	-	-	chalcone synthase
		112775981	-	ns	ns	
		112741589	-	-	-	chalcone synthase

		112796274	-	-	ns	
		112795670	-	-	-	chalcone synthase
		112715701	-	ns	ns	
		112741594	-	-	ns	
		112711206	-	ns	ns	
		112741559	-	-	-	chalcone synthase
		112722517	-	ns	ns	
		112796255	-	-	ns	
		112726613	+	ns	+	
		112741585	-	-	ns	
		112796265	-	ns	-	
		112695628	-	ns	ns	
		112734192	-	ns	ns	
		112695255	+	ns	ns	
		112796566	-	ns	ns	
		112796256	-	-	-	chalcone synthase
		112710078	-	ns	ns	
		112742455	-	ns	ns	
		112741855	-	ns	ns	
		112792033	-	ns	ns	
		112775424	-	+	ns	
		112796266	-	-	ns	
		112770735	-	ns	ns	
		112796258	-	-	-	chalcone synthase
		112697083	+	+	ns	
		112796278	-	-	-	chalcone synthase
		112710671	-	+	ns	
		112741574	-	-	ns	
		112727304	-	ns	ns	
		112741590	-	-	-	chalcone synthase
		112695653	ns	+	ns	
		112757529	ns	+	ns	
		112726445	ns	+	+	
		112796253	ns	-	ns	
		112740948	ns	-	ns	
		112755723	ns	-	ns	
		112741553	ns	-	-	
		112796277	ns	-	ns	
		112796259	ns	-	ns	
		112741581	ns	-	ns	
		112741570	ns	-	ns	
		112741580	ns	-	ns	
		112755722	ns	-	ns	
		112741542	ns	-	ns	
		112741543	ns	-	ns	
		112741586	ns	-	ns	
		112741591	ns	-	-	
		112796251	ns	-	ns	
		112758769	ns	-	ns	
		112736571	ns	-	ns	
		112796261	ns	-	ns	
		112796257	ns	-	ns	
		112741566	ns	-	ns	
		112733668	ns	ns	+	
Tropane, piperidine and pyridine alkaloid biosynthesis	adu00960	112796254	-	-	-	chalcone synthase
		112790504	-	-	-	chalcone synthase
		112720208	-	-	-	chalcone synthase
		112741555	-	-	-	chalcone synthase
		112741561	-	-	-	chalcone synthase
		112796273	-	ns	-	
		112801348	+	ns	+	
		112796275	-	-	ns	

		112740947	-	-	-	chalcone synthase
		112741589	-	-	-	chalcone synthase
		112796274	-	-	ns	
		112795670	-	-	-	chalcone synthase
		112741594	-	-	ns	
		112741559	-	-	-	chalcone synthase
		112796255	-	-	ns	
		112741585	-	-	ns	
		112801171	-	ns	ns	
		112796265	-	ns	-	
		112796256	-	-	-	chalcone synthase
		112796266	-	-	ns	
		112796258	-	-	-	chalcone synthase
		112697083	+	+	ns	
		112796278	-	-	-	chalcone synthase
		112741574	-	-	ns	
		112796915	-	ns	ns	
		112741590	-	-	-	chalcone synthase
		112778213	ns	-	-	
		112796253	ns	-	ns	
		112740948	ns	-	ns	
		112755723	ns	-	ns	
		112772978	ns	-	ns	
		112741553	ns	-	-	
		112796277	ns	-	ns	
		112796259	ns	-	ns	
		112741581	ns	-	ns	
		112741570	ns	-	ns	
		112741580	ns	-	ns	
		112755722	ns	-	ns	
		112741542	ns	-	ns	
		112715229	ns	-	ns	
		112741543	ns	-	ns	
		112741586	ns	-	ns	
		112741591	ns	-	-	
		112796251	ns	-	ns	
		112796261	ns	-	ns	
		112796257	ns	-	ns	
		112741566	ns	-	ns	
		112733645	ns	ns	+	
		112707518	ns	ns	+	
		112783205	ns	ns	+	
Biosynthesis of various plant secondary metabolites	adu00999	112802407	+	ns	+	
		112762412	-	-	-	fraxetin 5-hydroxylase
		112756986	+	+	+	S-adenosylmethionine synthetase
		112758002	-	ns	-	
		112750858	+	ns	+	
		112766831	+	ns	+	
		112695681	+	+	+	S-adenosylmethionine synthetase
		112696961	+	+	+	beta-glucosidase
		112705794	-	-	ns	
		112798030	-	-	-	pinoresinol/lariciresinol reductase
		112727916	-	ns	-	
		112717830	-	-	ns	
		112782427	-	-	ns	
		112710725	-	-	ns	
		112779478	-	-	ns	
		112697181	+	+	+	scopoletin glucosyltransferase
		112803923	+	-	ns	
		112711643	+	+	+	scopoletin glucosyltransferase
		112783185	+	+	+	beta-glucosidase
		112722671	+	+	+	S-adenosylmethionine synthetase

		112765003	+	ns	ns	
		112708410	-	ns	ns	
		112716851	+	+	+	beta-glucosidase
		112706619	-	ns	ns	
		112722947	+	+	+	beta-glucosidase
		112702200	+	ns	ns	
		112707447	+	+	+	S-adenosylmethionine synthetase
		112775183	ns	-	ns	
		112711180	ns	-	-	
		112783188	ns	-	ns	
		112779634	ns	-	ns	
		112710888	ns	-	ns	
		112779605	ns	-	ns	
		112716853	ns	-	ns	
		112737841	ns	+	+	
		112789968	ns	+	ns	
		112791984	ns	+	ns	
		112754842	ns	+	ns	
		112705587	ns	+	+	
		112802300	ns	+	ns	
		112736766	ns	+	+	
		112734146	ns	+	ns	
		112697063	ns	+	ns	
		112695481	ns	+	ns	
		112751027	ns	+	+	
		112754951	ns	+	ns	
		112695583	ns	+	ns	
		112741192	ns	+	ns	
		112758211	ns	+	ns	
		112783059	ns	+	ns	
		112740785	ns	+	ns	
		112802406	ns	+	ns	
		112788864	ns	+	ns	
		112766923	ns	+	ns	
		112783593	ns	ns	-	
Phenylpropanoid biosynthesis	adu00940	112782895	+	+	+	caffeoyl-CoA O-methyltransferase
		112715608	+	+	+	caffeoyl-CoA O-methyltransferase
		112707649	+	ns	+	
		112775852	-	ns	-	
		112740804	+	+	+	4-coumarate--CoA ligase
		112711957	+	+	+	cinnamyl-alcohol dehydrogenase
		112784679	+	+	+	caffeoyl-CoA O-methyltransferase
		112697465	+	+	+	caffeoylshikimate esterase
		112696779	+	+	+	5-O-(4-coumaroyl)-D-quinate 3'-monooxygenase
		112784538	+	+	+	caffeic acid 3-O-methyltransferase
		112789657	-	-	-	ferulate-5-hydroxylase
		112715717	+	+	+	caffeic acid 3-O-methyltransferase
		112795528	+	+	+	4-coumarate--CoA ligase
		112754637	+	+	+	5-O-(4-coumaroyl)-D-quinate
		112723608	+	+	+	caffeoylshikimate esterase
		112749801	-	-	-	4-coumarate--CoA ligase
		112738119	-	ns	-	
		112790259	-	-	-	coniferyl-aldehyde dehydrogenase
		112709716	+	+	+	shikimate O-hydroxycinnamoyltransferase
		112780096	+	+	+	shikimate O-hydroxycinnamoyltransferase
		112775398	+	+	+	cinnamyl-alcohol dehydrogenase
		112723541	+	+	+	4-coumarate--CoA ligase
		112714786	-	-	-	cinnamyl-alcohol dehydrogenase
		112741452	+	ns	+	
		112779679	+	ns	ns	
		112710403	+	ns	ns	
		112708172	-	ns	-	

112698284	+	ns	+	
112751186	-	ns	ns	
112737548	+	ns	ns	
112796200	+	ns	ns	
112782631	-	-	-	cinnamyl-alcohol dehydrogenase
112695881	+	ns	ns	
112696964	+	ns	ns	
112755082	-	ns	ns	
112716210	-	-	ns	
112696399	+	ns	ns	
112712613	+	+	+	phenylalanine ammonia-lyase
112792360	+	+	ns	
112750484	-	ns	ns	
112782318	-	ns	-	
112782699	+	ns	ns	
112771976	+	+	+	peroxidase
112749019	-	ns	-	
112802723	-	ns	ns	
112777165	+	+	ns	
112771695	-	ns	-	
112802159	-	ns	ns	
112741491	+	+	ns	
112754741	+	+	+	peroxidase
112748720	+	ns	ns	
112789622	+	ns	ns	
112796157	+	+	+	peroxidase
112800666	-	-	-	coniferyl-alcohol glucosyltransferase
112734573	+	+	ns	
112801342	-	ns	-	
112764966	-	ns	ns	
112737229	+	+	+	4-coumarate--CoA ligase
112776362	+	ns	ns	
112723657	+	ns	ns	
112754846	-	-	-	shikimate O-hydroxycinnamoyltransferase
112792364	+	+	ns	
112702704	+	ns	ns	
112747973	-	ns	-	
112803836	-	-	-	coniferyl-aldehyde dehydrogenase
112802156	+	ns	ns	
112720643	+	ns	ns	
112767040	+	ns	ns	
112711694	+	+	ns	
112738241	+	ns	ns	
112719905	+	ns	ns	
112712316	-	ns	ns	
112779615	+	+	+	phenylalanine ammonia-lyase
112754354	-	ns	ns	
112720314	-	ns	-	
112741451	+	ns	+	
112800604	+	ns	ns	
112801773	+	+	ns	
112702699	+	ns	ns	
112715722	+	ns	ns	
112755624	+	ns	ns	
112758023	+	ns	ns	
112722928	+	+	+	peroxidase
112697184	-	ns	ns	
112719871	+	ns	ns	
112723581	+	ns	ns	
112697826	+	ns	ns	
112796578	+	ns	ns	
112697395	+	ns	ns	

112782630	-	-	-	cinnamyl-alcohol dehydrogenase
112714787	ns	-	-	
112771440	ns	-	-	
112706825	ns	-	ns	
112696965	ns	-	ns	
112745809	ns	-	ns	
112727631	ns	-	ns	
112796154	ns	-	ns	
112728188	ns	-	ns	
112765227	ns	-	-	
112758738	ns	+	ns	
112696127	ns	+	ns	
112765574	ns	+	ns	
112723543	ns	+	ns	
112748390	ns	+	+	
112785078	ns	+	ns	
112705729	ns	+	+	
112799024	ns	+	ns	
112715276	ns	+	ns	
112799008	ns	+	+	
112743806	ns	+	ns	
112800509	ns	+	ns	
112797152	ns	+	ns	
112720495	ns	+	ns	
112696397	ns	+	+	
112789302	ns	+	ns	
112758044	ns	+	ns	
112789303	ns	+	ns	
112702311	ns	+	ns	
112741448	ns	+	ns	
112715071	ns	+	ns	
112793739	ns	+	ns	
112720490	ns	+	ns	
112698272	ns	+	ns	
112696962	ns	ns	-	
112749000	ns	ns	-	
112765954	ns	ns	-	
112744093	ns	ns	+	

"+": Positive, compared to the Control, the gene expression level was increased; "-": Negative, compared to the control, the gene expression level was decreased; "ns": not significant, compared with the Control, the gene expression was not significant.

Table S6 Soil physicochemical properties.

Soil properties	Classification / Value
Soil type	Brown soil
Soil texture	Clay loam soil (sticky grains 36.6 %, powder grains 32.5 %, sand grains 30.9 %)
Electrical conductivity (mS/m)	103.00 ± 3.00
Field capacity (%)	23.73 ± 0.97
Temperature (℃)	26.03 ± 0.27
Cation exchange capacity ($cmol^+$ /kg)	15.00 ± 4.25
pH	6.15 ± 0.06
Organic matter (g/kg)	12.68 ± 0.13
Avaliable N (mg/kg)	96.61 ± 1.24
Avaliable P (mg/kg)	352.05 ± 10.03
Avaliable K (mg/kg)	111.29 ± 11.29
